# Smart Air Quality Monitoring IoT-Based Infrastructure for Industrial Environments

**DOI:** 10.3390/s22239221

**Published:** 2022-11-27

**Authors:** Laura García, Antonio-Javier Garcia-Sanchez, Rafael Asorey-Cacheda, Joan Garcia-Haro, Claudia-Liliana Zúñiga-Cañón

**Affiliations:** 1 Instituto de Investigación para la Gestión Integrada de Zonas Costeras, Universitat Politècnica de València, 46730 Valencia, Spain; 2 Department of Information and Communications Technologies, Universidad Politécnica de Cartagena, 30202 Cartagena, Spain; 3 Research Group COMBA I+D, Universidad Santiago de Cali, Cali 760035, Colombia

**Keywords:** air quality monitoring, particulate matter, polluting gas, machine-learning

## Abstract

Deficient air quality in industrial environments creates a number of problems that affect both the staff and the ecosystems of a particular area. To address this, periodic measurements must be taken to monitor the pollutant substances discharged into the atmosphere. However, the deployed system should also be adapted to the specific requirements of the industry. This paper presents a complete air quality monitoring infrastructure based on the IoT paradigm that is fully integrable into current industrial systems. It includes the development of two highly precise compact devices to facilitate real-time monitoring of particulate matter concentrations and polluting gases in the air. These devices are able to collect other information of interest, such as the temperature and humidity of the environment or the Global Positioning System (GPS) location of the device. Furthermore, machine learning techniques have been applied to the Big Data collected by this system. The results identify that the Gaussian Process Regression is the technique with the highest accuracy among the air quality data sets gathered by the devices. This provides our solution with, for instance, the intelligence to predict when safety levels might be surpassed.

## 1. Introduction

Air quality is one of the main aspects to consider in environmental impact assessment. It not only affects the environment but also has an effect on the health of the people living and working near industrial activities. Industrial spaces are particularly susceptible to air contamination due to the manipulation of chemical components and processes that emit polluting gases and small particles into the air. These types of particles are made up of a complex mixture of solid, solid and liquid, or liquid particles of organic and inorganic substances suspended in the air [1]. They can penetrate the respiratory tract, reaching greater depths the smaller their size [2]. The WHO states that particulate matter is affecting the world’s population more than any other type of pollutant. In addition, it recommends not exceeding the levels of particles specified in Table 1 [3]. Therefore, polluting gases that could potentially harm human health are also present in the air. These gases are often monitored together with particulate matter levels to establish air quality indexes, such as the European Air Quality Index [4] that provides an air quality evaluation ranging from good to extremely poor based on the data gathered from over 3500 stations and following the EU air quality directives [5]. Sulfur dioxide (SO${}_{2}$), nitrogen dioxide (NO${}_{2}$), ozone (O${}_{3}$), and carbon monoxide (CO) are among the most harmful substances. Table 2 shows the threshold values that the WHO recommends not exceeding [6]. As a consequence, industries are requesting ways to track and control their emissions in real time to avoid facing penalties for exceeding the limits established where they operate.

Air quality is usually monitored using precision stations deployed at specific locations, many of which are integrated into (official) meteorology monitoring stations. However, as interest in monitoring air quality to improve health and implement better policies has increased, the development of portable and affordable devices has multiplied. Specifically, there are many proposals in the context of Smart Cities for indoor and outdoor air quality monitoring [7]. These types of systems are usually deployed under the Internet of Things (IoT) umbrella [8], where devices are used in a variety of locations. The data gathered can be processed to map a city’s air quality [9] or provide the air quality history of a location. Other proposals include devices such as pUAV to monitor air quality from different areas [10]. These devices can communicate with others through wireless connections or connect to the Internet with a cabled Ethernet connection to forward data to the server for storage and processing. Among the available wireless technologies, WiFi, ZigBee, or LoRa are popular choices for IoT air quality monitoring. Monitored data analysis has also evolved to include Artificial Intelligence (AI) techniques. Machine learning, as a part of the solutions AI provides, is suitable to perform predictions and estimations of air quality [11]. Furthermore, environments such as industrial facilities may need to adopt certain standards to ensure interoperability with other systems and devices, for example, the INSPIRE specifications based on infrastructures for spatial data [12].

This paper presents an air quality measurement architecture comprised of devices capable of measuring polluting gases and suspended particulate matter. To facilitate the installation of these devices so that they are totally independent of the electrical network, an anti-vandalism structure with a solar panel on the top and housing, an auxiliary battery to provide continuous power has been designed. In addition, a LoRaWAN network has been designed, implemented, and deployed, through which the devices communicate with a server that processes and stores the data captured in databases for further analysis. An alert system has also been developed with email alerts and/or instant message alarms. We have designed and implemented software modules to communicate our solution with OPC systems. These systems are widely used in the industry to control pPLC and the information exchanges between their systems. Moreover, the Big Data generated has been extensively analyzed using several machine learning techniques to determine which is the most accurate one. Therefore, it is possible to forecast trends of future data and predict, for instance, inadmissible pollution levels. The combination of the above functionalities results in a robust air quality measurement architecture that can be fully integrated with the systems traditionally used by industry. This makes it an ideal industry focused solution for real-time air quality monitoring. Specifically, the proposed system would benefit industries working with soils, stones, grains, or other materials that can produce particulate matter as well as industries susceptible to high levels of NO${}_{2}$, O${}_{3}$, SO${}_{2}$, and CO.

The rest of the paper has been organized as follows. Section 2 presents the related work. In Section 3, the developed infrastructure is detailed. Section 4 describes the design of the monitoring devices. Section 5 discusses the obtained results. Finally, Section 6 concludes.

## 2. Related Work

Insufficient air quality can lead to severe health problems, but precision air quality monitoring devices may be costly and difficult to deploy in a variety of settings. As a result, several studies have been conducted to determine the correlation between more affordable sensors and precision air quality stations. Evangelos Bagkis et al. analyzed in [13] the performance of an air quality monitoring device compared to a reference station. Seven machine learning algorithms were employed to model a correction factor. The results showed that variations in the meteorological conditions affected the quality of the data. Among all the techniques tested, the Convolutional Neural Network (CNN) obtained the best overall performance. Furthermore, the average of several estimators improved the metrics. Byoung Gook Loh et al. used Web Query and Machine Learning to calibrate a device for PM${}_{2.5}$ particulate matter monitoring [14]. The algorithms employed as regression models for the calibration were k-nearest neighbors, Extreme Gradient Boosting, Support Vector Machine, and Random Forest. Stratified k-fold cross-validation was applied to evaluate the performance. Results showed that the best performance was obtained by the Extreme Gradient Boosting algorithm.

As air monitoring devices have become more affordable, and health concerns about poor air quality have increased, interest in deploying air quality monitoring systems in different environments has grown. Andrew Rebeiro-Hargrave et al. developed in [9] a system for urban air quality monitoring. Their system can generate history graphs and pollution maps from the information gathered. The devices are portable and low-cost and can monitor air pollutant gases (O${}_{3}$, CO, and NO${}_{2}$) and PM${}_{2.5}$ particles. The system was tested in Helsinki, and a pollution profile of the city was created. It was presented as a tool to generate policies to improve the city’s air quality. Aditiyo Hermawan Kuncor et al. designed in [15] an air quality monitoring system based on IoT that was deployed and tested in the city of Tasikmalaya. CO, O${}_{3}$, and CH${}_{4}$ were monitored using MQ-7, MQ-131, and MQ-4 sensors, respectively. Arduino microprocessors were employed to obtain the data from the sensors and forward them through the Internet to be visualized in real-time. Steven J. Johnston et al. presented in [16] a system for air quality monitoring. The devices were equipped with four PM sensors and LoRaWAN transceivers. Tests were performed in the city of Southampton in the UK. The results proved that the system performed correctly on a city scale. Furthermore, some of the low-cost sensors were suitable for monitoring particles and detecting trends. These types of systems for air quality monitoring are usually deployed as Wireless Sensor Networks (WNS), such as the one presented by Patricia Arroyo et al. [17]. Data were gathered from the ZigBee nodes and forwarded to the server through the gateway. Cloud computing systems stored, processed, and monitored volatile organic compounds, such as benzene, xylene, ethylbenzene, and toluene. Data were processed using Support Vector Machine and a backpropagation learning algorithm. The results showed good behavior in obtaining concentrations of volatile organic compounds. The work by Ivan Popović et al. proposed a framework for air quality monitoring in urban environments [18]. The system was designed with a layered architecture based on fog computing supporting real-time operation to perform activities such as fault diagnosis and automatic reporting. The processing performed on the sensors was presented as microservices located on the different layers of the architecture. The system was deployed and tested in a time frame of six months, monitoring O${}_{3}$, CO, CO${}_{2}$, SO${}_{2}$, NO, and NO${}_{2}$, as well as PM${}_{1}$, PM${}_{2.5}$, and PM${}_{10}$. Meteorological parameters, such as air temperature, humidity, and pressure were also monitored. The results corroborated good system performance.

In addition to urban environments, indoor and industrial environments are also considered for air quality measurement deployments. JunHo Jo et al. presented in [19] an indoor air quality monitoring system able to measure CO, CO${}_{2}$, VOC, and aerosol concentrations, in addition to air temperature and humidity. The data were forwarded to the server in real time through LTE and could be accessed through a web server or a specifically developed application. The system was tested at Hanyang University, and the results showed the prototype implementation and data collected from the application. Judith Molka-Danielsen et al. in [20] studied the deployment of an air quality monitoring system in industrial environments, specifically, a logistics shipping base. The authors discussed how to process the obtained data to evaluate the impact of high CO${}_{2}$ levels in an industrial workplace. They stressed the importance of the correct transformation of data to facilitate their visualization. Furthermore, the authors suggested using smart closed-loop systems that could detect spikes of potentially harmful polluting gases and trigger actuators to provide more ventilation.

The popularity and effectiveness of AI has led to the introduction of machine learning techniques in air quality monitoring systems. C. Amuthadevi et al. used different machine learning approaches to develop an air quality monitoring model [11]. The selected machine learning methods were Non-Linear Artificial Neural Network (ANN), Neuro-Fuzzy, Statistical Multilevel Regression, and Deep Learning Long-Short-Term Memory (DL-LSTM). To determine the accuracy of the different methods, the RMSE (root-mean-square error), R${}^{2}$, and MAPE parameters were used. Outcomes showed that DL-LSTM presented the best result among the tested methods. Dixian Zhu et al. employed machine learning to develop a forecasting model for air quality [21]. They used a refined model with regularization to enforce the prediction models. MTL, nuclear norm regularization, Frobenius norm regularization, and l${}_{2,1}$ -norm regularization were compared. The results of the experiments proved the efficiency of their proposed method. Similarly, Naomi Zimmerman et al. [22] employed machine learning to create calibration models to improve the performance of low-cost sensors for air quality monitoring. The results of testing univariate Linear Regression, Random Forests, and Empirical Multiple Linear Regression showed Random Forests to be the one that enabled low-cost sensors to meet the requirements of the US EPA Air Sensors Guidebook. In addition, differences in NO${}_{2}$ concentrations were found in less than 1.5 km. Finally, other works, such as [23] dealt with data acquisition and IoT communication architecture from a general perspective.

The existing proposals have been developed considering the environment of the deployment without taking into account any standards in device design apart from communication protocols. This could be due to a lack of standards for certain environments. However, considering the existing standards in industrial environments it is important to ensure the integration, interoperability, and scalability of feasible solutions. Therefore, the solution proposed in this work integrates high-quality components calibrated with precision sensors. We adopt the OPC standard, which is widely used in industrial environments. Furthermore, analyzing the acquired air quality data with machine learning techniques has contributed to determining the best algorithm for processing air quality data in industrial environments and predicting trends for future datasets.

## 3. Architecture Description

In this section, the proposed system architecture for air quality monitoring in industrial environments is described.

**Figure 1:**
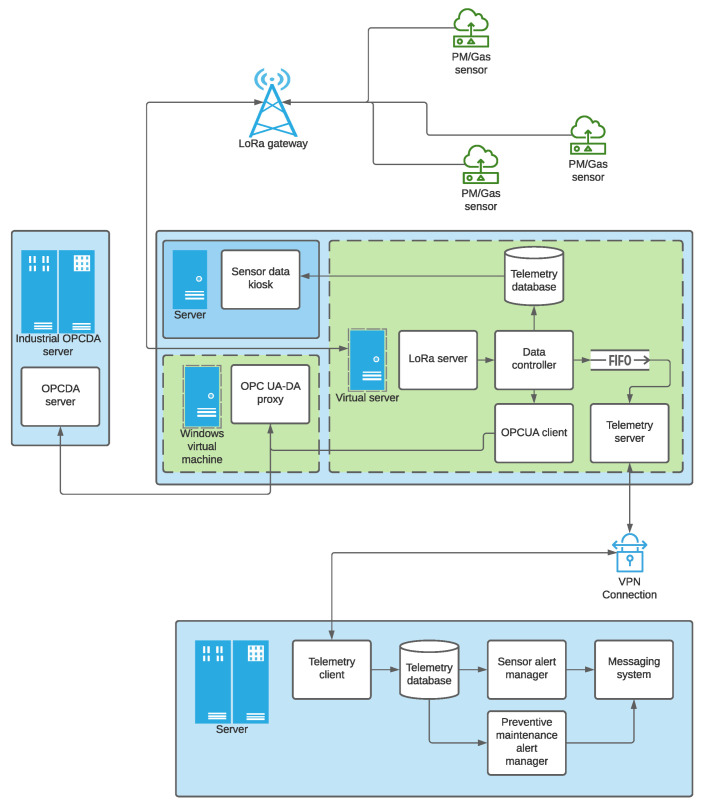
System architecture.

Figure 1 shows the general scheme of the developed infrastructure. The goal is to plot air quality for end-clients from the data collected by a series of sensors. The acquired data is forwarded by the devices using LoRa communication technology on the 868 MHz frequency band, as stated in the LoRaWAN specification for deployments in Europe [24].

The server has a data management system in charge of storing data in an InfluxDB database [25]. This type of database stores time series of data and manages the huge amounts of data generated by the devices, applications, and infrastructures, providing a timestamp for each of them when stored. This is useful for their later representation in graphs using the Grafana software. Grafana is a web server that depicts data time series in graph format [26]. This web server represents the evolution (over time) of the particulate matter or polluting gases and the internal parameters of the devices, such as communication link quality levels, allowing the visualization and supervision of the deployment. In this way, any device connected to the internal network of a client company can display the data. Furthermore, different types of data sources can be configured in Grafana. In this proposal, the InfluxDb data base and Prometheus monitoring system were configured to visualize the state of the servers and the operating services. The server integrates an OPCUA (OPC Unified Architecture) server and, if necessary, there is also a virtual machine in Windows OS where a proxy can obtain the data stored in the OPCUA server and send it to the OPCDA (OPC Data Access) client company’s server. The use of Windows OS is required since OPCDA uses Microsoft DCOM protocol.

The data reaching the OPCDA server is then forwarded to an external server through a VPN to facilitate a secure connection. A copy of the data is stored on this server as well, and Grafana can represent the data from any device with an Internet connection since it has its own public IP address that the client can access. This server also has an alert system. As a basic service, Grafana’s integrated default warning system can be used to inform the user when abnormal levels of contamination are detected. As an advanced service, the *Prometheus Alertmanager* tool will notify the network manager of any failure detected in any of the provided services. In the following subsections, all these components are described in detail.

### 3.1. Network Deployment

A LoRaWAN network has been developed including the different gateways required to send the received data to a server through the network. LoRaWAN is a Medium Access Control protocol for wide area networks built on the LoRa modulation. It enables low-power devices with applications connected to the Internet to communicate through long-range wireless communication links. LoRa is a Wireless modulation technology robust against electromagnetic disturbances and able to provide long-distance data transmissions. It is suitable for telemetry applications where small data packets are transmitted using low bitrates. Its built-in features make LoRa an excellent technology choice for low-power sensors. It can be used with different unlicensed frequency bands, such as 915 MHz, 868 MHz, and 433 MHz [27], depending on the region where the devices are located. In this proposal, the suitable frequency band for Europe is the 868 MHz unlicensed band.

**Figure 2:**
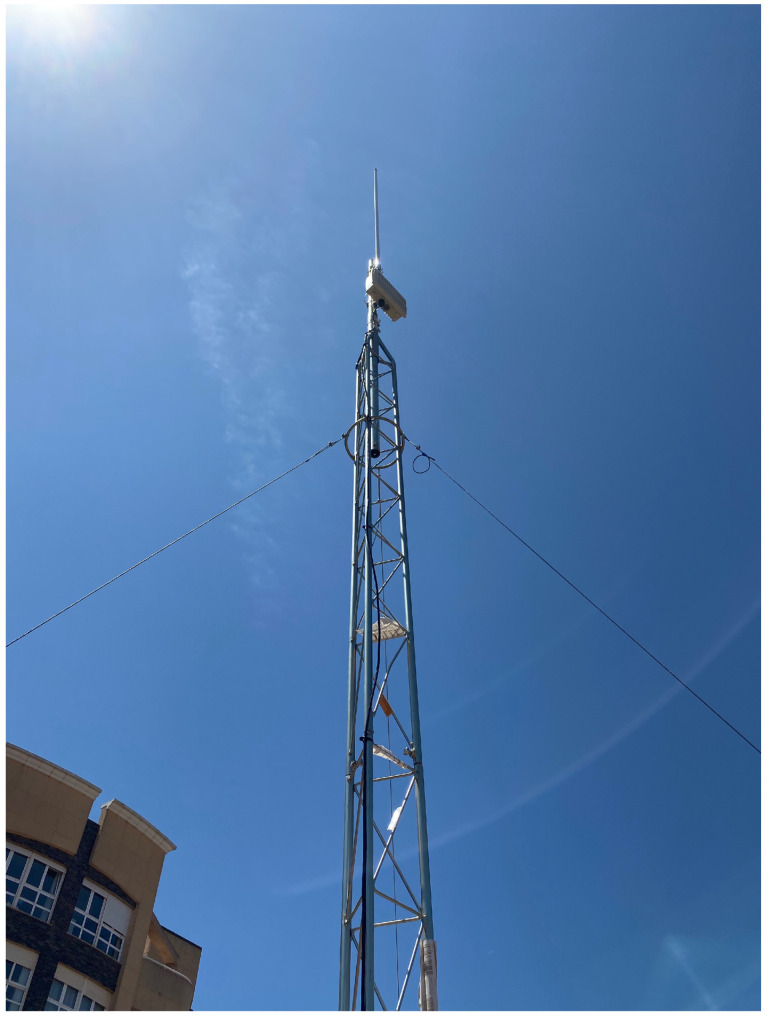
Antenna and Gateway.

An outdoor gateway able to offer radio coverage of approximately 10 km in rural areas and 1 km in urban areas has also been used. This gateway provides bidirectional communication between the devices connected to the network and the server, which is connected to the gateway through an Ethernet connection. Figure 2 illustrates the antenna and the gateway we have deployed and installed for this work. The server is equipped with Chirpstack, which is a network server for LoRAWAN networks. It offers a web interface that easily configures new gateways, defines different types of applications for different uses, and adds the devices required for each application [28].

These devices, called microcontrollers, are responsible for data acquisition and their transmission to the network. The LoRa class establishing the form of communication must be configured in each of the devices. The LoRaWAN specification defines three classes of devices: class A, class B, and class C [27]. Our devices can able to operate as class A or C. The differences between the classes reside in the time the receive windows are open, which is two times for class A and constantly open for class C [29]. These receive windows enable communication from the gateway to the devices. However, the longer the receive window is open, the higher the energy consumption of the device. As a result, class A devices are more energy efficient and are usually powered by a battery [27]. For this reason, the microcontroller in this proposal has been configured as a class A device.

LoRa provides two authentication methods for devices: Over-The-Air-Activation (OTAA) and Authentication By Personalisation (ABP). The devices have a unique identifier known as *DevEUI*, which is defined by the manufacturer. However, to identify the device and all the communications coming from this device in a LoRa network, a not necessarily unique identifier is used. It is known as *DevAddr*. When the activation process starts, regardless of whether OTAA or ABP is employed, a *DevAddr* is assigned to the device. For ABP, the device has a *DevAddr* and static session keys stored in its memory. Therefore, even a network activation process is not necessary. In the case of OTAA, the device must initiate a login process to the network, and the *DevAddr* and the keys change as a new session is established [30]. For this proposal, both authentication methods have been used. However, ABP was prioritized. This is because OTAA must have a high-quality connection to ensure that the data packets coming from the gateway, which are necessary to activate the device, are received. This could be a problem for devices located in areas with limited radio coverage.

#### MQTT Server Configuration

An MQTT server is a lightweight protocol in which messages have a topic. The body of the message receives the data from the Chirpstack server. The data is then forwarded through a device to a broker that redirects them to the subscribers of the topic of the message. The subscribers can only receive messages with the topic they are subscribed to [31].

**Figure 3:**
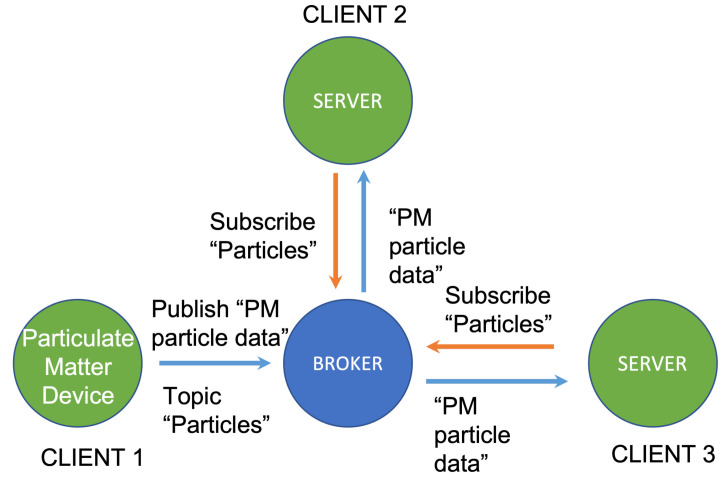
Diagram of the particulate matter device operation.

Figure 3 illustrates an example of an MQTT operation, where the particulate matter measuring device publishes a message with the topic “particles” and the broker re-sends it to the two servers subscribed to that topic. In our proposal, the Chirpstack server relays the data received through LoRa to a broker located within the server itself.

A series of scripts related to the subscribed topics of interest has been developed in Python. These topics include: (i) link quality data encompassed within a topic bridging information, like RSSI (Received Signal Strength Indicator) or SNR (signal-to-noise ratio), and (ii) environmental pollution data received from the sensors, which is included in the topics of each application configured in the Chirpstack server. When data reaches the MQTT server, these scripts decode, analyze, and store them in different InfluxDB databases. Redundant databases facilitate that the data be stored certainly in our system.

### 3.2. OPC Standard

To develop an operational industrial infrastructure to monitor air quality, the devices must consider the conditions of industrial environments both in hardware and software design. Specifically, regarding the software, the system has been integrated into the open-source OPC communication standard, which is widely used to monitor industrial processes among pieces of equipment from different manufacturers.

In the case of OPC DA, the client accesses data from the server locally or through an Ethernet connection. Moreover, the server can simultaneously read and write commands from the client. This allows for three methods of accessing the data from the OPC server: in synchronous mode, asynchronous mode, or subscription mode [32].

The OPC UA [33] is a more modern version that integrates all the functionalities of the OPC specifications into one working environment. Apart from adding the classic operations, it includes other functionalities, such as finding available OPC servers on the network, managing important notifications according to the client’s requirements, and hierarchically representing the data, among other things. It is independent of the platform; thus, it can be run on any device with any operating system. It includes important security improvements such as identifying both clients and servers through X509 certificates and requesting client authentication to gain access to particular applications. It also allows new functionalities to be added without disrupting the existing applications, which ensures smooth operating with newly developed systems.

The Linux server used in this proposal is hosted in an OPC UA server developed in Python. Clients can access this server to receive their data. If sending the data to an OPC DA server is required, a proxy is developed in a Windows machine to obtain the data from the OPC UA server and forward them to the OPC DA server. Windows was used because this operating system is the only one that permits the use of DCOM, which is a Microsoft-developed technology needed for OPC DA communications. To develop the proxy, the OpenOPC library from Python was used.

### 3.3. Sending Alerts

Alerts are sent in two ways. The first one sends alerts from the Grafana server. These types of alerts inform the client of high pollution levels, low battery levels, connection loss with the devices, or malfunctions. One of the advantages of using Grafana to send alerts is the convenience of attaching the panels where the data time series are represented. This allows the client to visualize the evolution of the pollution levels leading to the alert in their email. Grafana also permits alert transmission through the instant messaging application Telegram.

Prometheus software has been used to monitor the servers and the offered services. It is an open tool for monitoring and sending alerts about the state of the devices and services. *Alertmanager* is one of the integrated services available in Prometheus. It provides a web interface where the metrics and the state of the devices are represented called *Node Exporter*, which monitors the server where the Prometheus software is installed. One of the Prometheus tools used in the system presented in this paper is the Blackbox Exporter. This tool monitors the devices using protocols such as HTTP, TCP, or ICMP. It can provide multiple metrics such as the general state of the device, response time, and redirection information [34]. Prometheus has numerous advantages. One of them is the possibility of performing powerful queries to obtain the stored time series data and use them to generate graphs, tables, or alerts. It also offers varied visualization options, including its own web interface and the integration of the data in Grafana panels. Alerts can be sent as well and it can also be integrated with third-party applications [35]. In the case of the Prometheus *Alertmanager* extension, which is used to send alerts to network administrators, the possibility of dispatching emails to inform about system crashes is available.

## 4. Design of the Monitoring Devices

In this section, the designs of the suspended particulate matter measuring device, polluting gas measuring device, structure that protects the devices, and powering system are presented in detail. Note that the company manufacturing these devices is denoted as Qartia Smart Technologies [36].

### 4.1. Suspended Particulate Matter Measuring Device

**Figure 4:**
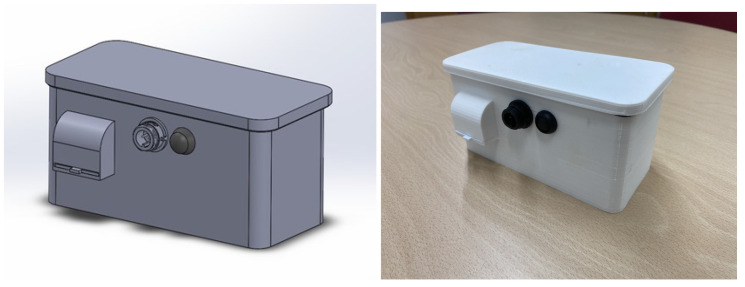
Polluting particle metering device.

The development of the device to measure particulate matter in suspension began with the integration of its different components. Inside the device, which can be seen in Figure 4, there is a suspended particulate matter measuring sensor that can measure particles in the range of 0.3 μm to 40 μm. It can be configured to measure any size particles within that range. In this case, the device has been configured to measure PM${}_{10}$, PM${}_{2.5}$, and PM${}_{1.0}$ particles. In addition, the device has a temperature and humidity sensor, a GPS receiver, and a microcontroller. The microcontroller reads the data from the sensors, processes them, and sends them to the LoRaWAN network. It has been programmed using the *MicroPython* programming language, which is an efficient and simple implementation of the Python 3 language with a subset of Python libraries, optimized for use in microcontrollers [37].

Programming in *MicroPython* is very simple since the code is loaded into the internal memory with a program designed for a specific type of microcontroller. Communication with the microcontroller is usually set by emulating a serial interface [38], and the programming is carried out through a terminal application on a computer. We have configured the device to send data every five minutes, which is a reasonable value for industrial processes. These data are the average particle concentrations registered since the previous data were sent, as well as the temperature, humidity, and GPS positioning. To communicate with the different sensors connected to the microcontroller, *MicroPython* has a library capable of handling different communication interfaces [37], such as SPI, used in communication with the particle sensor, and UART, for communication with the GPS or I2C receiver needed to read data from the temperature and humidity sensors.

Inside the device, there is a charge controller that manages the recharging process of an internal high-capacity lithium battery. It has approximately two-days’ autonomy in case it is disconnected from the power grid or the auxiliary battery. When an occasional measurement in a concrete area is needed, our solution can be portable. The device can be moved to the location required. Furthermore, a casing has been designed keeping in mind that the device must withstand adverse weather conditions. It includes air input and output on both sides for the particle sensor. A nozzle faces downwards to ensure that the device is protected and the particle concentrations are correctly monitored.

**Figure 5:**
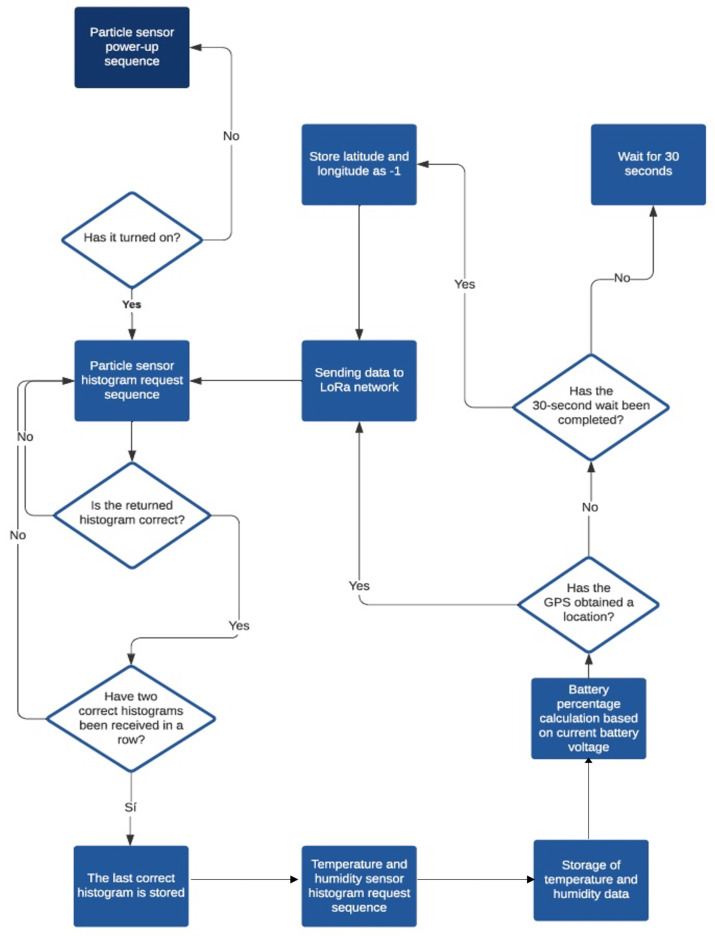
Flow diagram of the operation of the particulate matter measuring device.

The flow diagram of the software developed for this device is represented in Figure 5. After pressing the *ON* button, placed on one side next to the charge connector, the device begins the boot up sequence of the integrated particle sensor. Then, the request for the histogram is initiated and an average of the particle concentration data from the previous 5 min is obtained. Then, the device collects the temperature and humidity data and calculates the percentage of remaining battery. Next, the device finds the GPS location, which is only acquired in open spaces. All this information is sent to the LoRaWAN network. After that, the histogram request sequence starts again to continue running the software in a loop.

### 4.2. Polluting Gas Measuring Device

**Figure 6:**
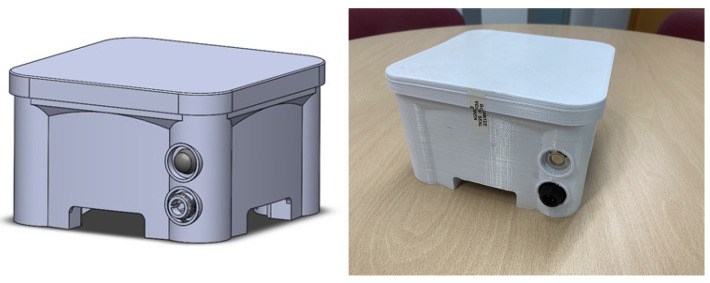
Polluting gas measuring device.

The development of this device also began with the integration of its different components. It (see Figure 6) was designed to measure four different types of polluting gases. In this case, the device can detect concentrations of four of the most dangerous gases found in the air: SO${}_{2}$, NO${}_{2}$, O${}_{3}$, and CO${}_{2}$. SO${}_{2}$ is released from coal and oil combustion, and it can lead to respiratory diseases or even death. NO${}_{2}$ is produced from road traffic and other fossil fuel combustion processes. It contributes to acid rain and can lead to pulmonary irritation, among other health problems. O${}_{3}$ originates from the chemical reaction between sunlight and the pollutants from vehicles and industries. It can lead to breathing difficulties, respiratory infections, or premature death. Lastly, CO${}_{2}$ is released in the combustion of wood, oil, and natural gas and has been linked to headaches, breathing difficulties, loss of consciousness, and even death [39].

The sensors react to gases generating voltage levels on their electrodes. These levels must be measured to calculate the gas concentration in the air. The measurement is performed by two electrodes: a working electrode and an auxiliary electrode. This compensates for the errors caused by the effects of ambient temperature and humidity. The aim is to obtain the equivalence between the voltage levels and the accurate concentration of each of the gases in μg/$m^{3}$. The calibration of the sensors was twofold. On the one hand, all the sensors were first calibrated under laboratory conditions. Secondly, a second calibration of all sensors in working conditions was made (outdoors). To do this, sensor devices were measuring during several weeks together with an official air quality station belonging to the administration. The results of both measurement systems were compared, and the measurements of our devices were adjusted using a linear regression. This second calibration allowed us to take into account the impact of interfering gases on our sensors without laboratory conditions.

The device includes analog-to-digital converters to obtain digital values from the voltage measured at the electrodes of the sensors. Then, the microcontroller integrated into the device reads these values and sends them through LoRa to the server to carry out postprocessing actions on the data. Similar to the particulate matter measuring device, the pollutant gas measuring device integrates a GPS and temperature and humidity sensor which, in this case, are necessary for postprocessing since the gas sensors are even more sensitive to temperature and humidity. In addition, this device includes a charge controller and a battery with enough capacity for three days of continuous use.

**Figure 7:**
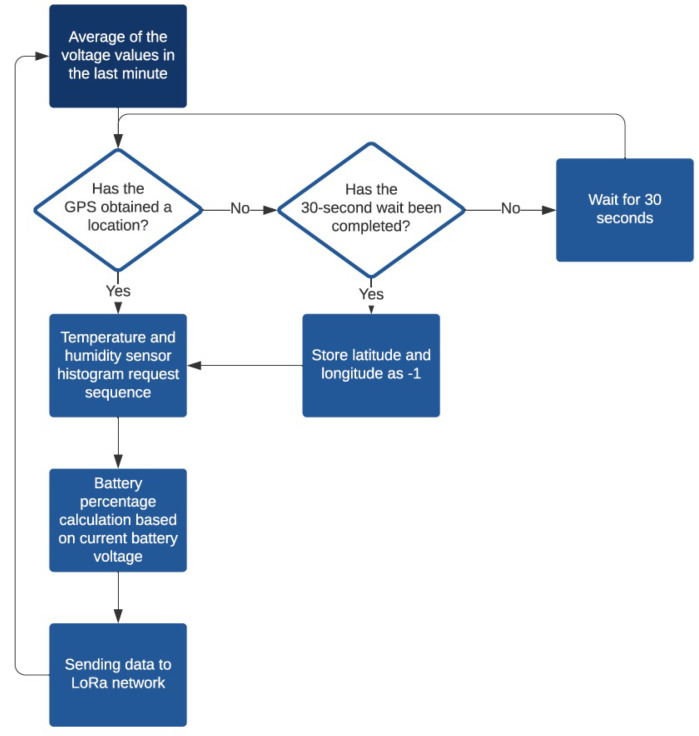
Flow diagram of the pollutant gas measuring device operation.

The software developed for this device reads the voltage values of the main and auxiliary electrodes of the gas sensors for 1 minute, carrying out one measurement per second and calculating the average value. This value is stored to later be forwarded to the server. After this, the GPS location of the device is attained and, lastly, the device reads the current temperature and humidity. Once all the necessary data are collected, they are dispatched to the LoRaWAN network for postprocessing and storage at the server. The flow chart of the operation of the device is presented in Figure 7.

The device was calibrated by obtaining voltage samples from the two electrodes of each of the sensors for a long time window (one month) with the device placed near an official station with highly calibrated gas sensors. Once the necessary samples were obtained, the data were stored in a *csv* format. This *csv* format includes hourly averages of the voltage data from the four sensors’ working and the auxiliary electrodes, ambient temperature and humidity data at the time the sample was taken, and the gas concentrations from the official station. The data from the official station is public and updated every hour with the average concentration of the previous hour.

The device was calibrated using Python and the Linear Regression method, which is one of the most well-known algorithms in machine learning. Linear Regression uses an equation to obtain a predicted value from the input data. A coefficient is assigned to each input value. When this coefficient is zero, the input value does not influence the result of the prediction [40]. To calculate the coefficients of the Linear Regression algorithm, data from the official station were used as the expected result. The voltage values from the electrodes and the ambient temperature and humidity obtained at the time of taking these samples were used as input values to calibrate each device. As a result, different adjustment coefficients were generated for each of the gas sensors. These coefficients must be multiplied by the input data to obtain the gas concentration in μg/$m^{3}$.

Note that measurements from other sensors were also considered since there is cross interference among them. Therefore, a sensor positioned to measure the concentration of a specific gas may react to the presence of other gases, producing undesirable voltage in its electrodes.

The results of this calibration process were highly satisfactory. Concentration values very similar (up to around 90%) to those of the official station were obtained. Therefore, our device is a good option as a small and affordable solution to measure the concentration of gases and particulate matter in the air of industrial environments.

### 4.3. Powering and Structure

One of the main objectives of this project is to create a structure that accommodates both devices, providing them with complete independence from the power grid. For this reason, the structure includes a solar panel at the top. This facilitates installation since the structure need only be placed in the desired location and the devices installed inside. However, it is necessary to previously verify that there is enough radio coverage for the devices to establish a connection to the gateway.

Some factors, such as deploying the solar panel inclined and facing south were considered. Moreover, the structure includes a large battery that can store the energy provided by the solar panel and provide the necessary weight to keep the structure stable without anchoring it to the floor, although the latter is recommended for improved safety. Both the battery and the solar panel controller are placed inside a watertight box in the lower part of the structure. The cables from the solar panel are connected to the controller through the lower part of the box. The power cords of the two devices monitoring air quality are also connected to the controller using USB ports through the lower part of the box. The controller is connected to the battery.

**Figure 8:**
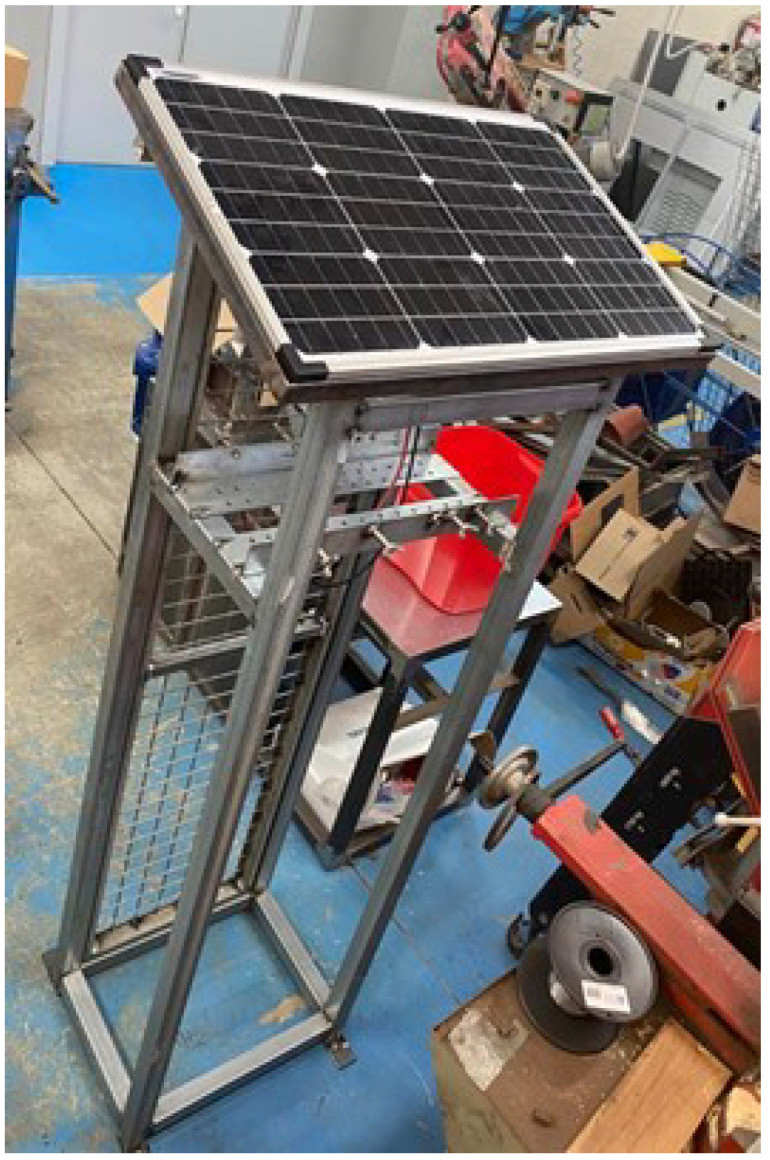
Storage structure for the air quality monitoring sensors in its development stage.

The solar panel is placed on the top of the structure protecting the suspended particulate matter measuring device and the polluting gas measuring device. This structure is encased in a metal grid with a door. The structure and solar panel are shown in Figure 8.

## 5. Results

This section presents the graphical interface and the alert features of our system, as well as the machine learning study determining the best prediction-making algorithm based on the data obtained from the sensors of the different devices discussed in the previous sections. These predictions permit early detection and report alerts about dangerous levels of gases or particulate matter.

### 5.1. Graphical Interface

**Figure 9:**
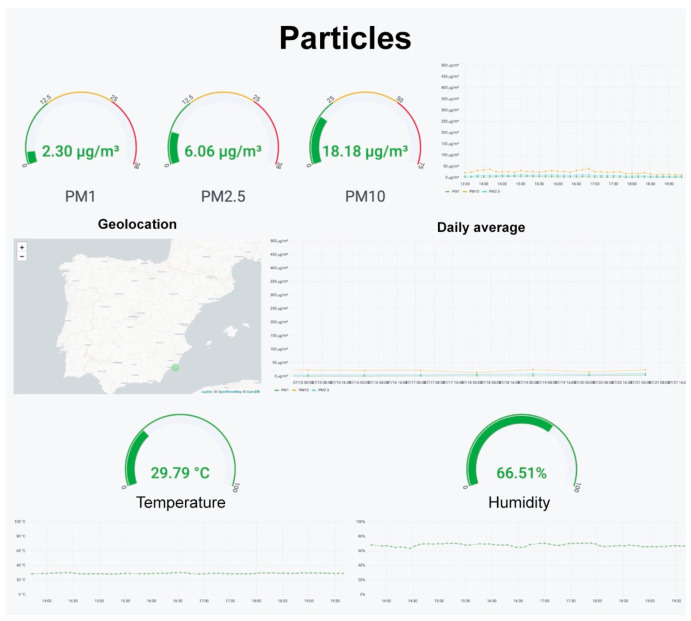
Visualization panel for the particulate matter concentrations in the air.

To visualize the data, Grafana panels were used. These panels show the information stored in the InfluxDB database sorted into different sections. The panel shown in Figure 9 shows how the information about particulate matter in the air is organized. It has been designed to show the average concentration of the previous half hour on the upper left part of the panel. There are also warning indicators that vary in color as concentrations increase. For example, the PM${}_{10}$ display is green if it does not surpass the 25 μg/$m^{3}$ threshold, yellow if the concentration ranges from 25 to 50 μg/$m^{3}$, and red when it exceeds 50 μg/$m^{3}$. These values are based on the WHO’s recommendations for annual and daily measurements, as indicated in Section 1. In the case of PM${}_{2.5}$, the same criteria was applied. Lastly, for PM${}_{1}$, the same values as PM${}_{2.5}$ were used since the WHO does not provide any recommendation.

The graph on the upper right part of the panel shows the evolution of particulate levels. The graph with the daily average of the previous seven days is displayed below. The central left part shows the location of the device, forwarded by the integrated GPS. To do this, the *WorldMAp* extension in Grafana must be installed. The real time temperature and humidity values and the graphs representing their evolution are located on the lower part of the panel.

**Figure 10:**
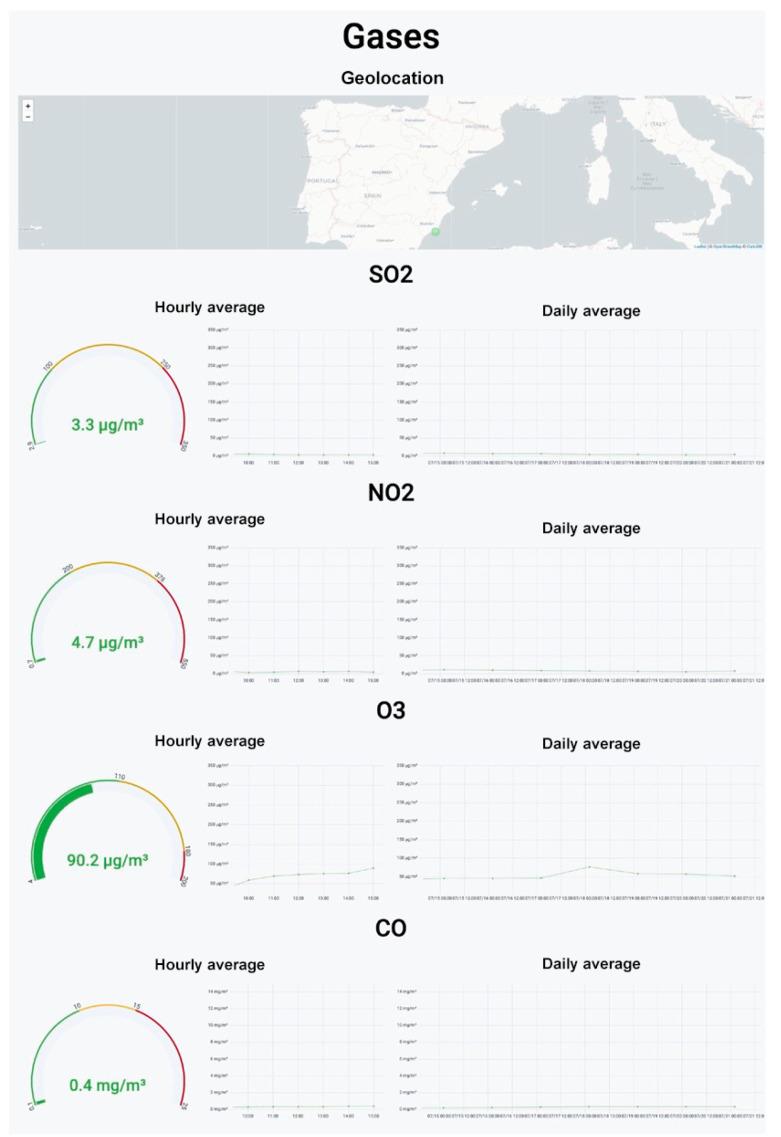
Visualization panel for concentrations of the four gases in the air.

Figure 10 shows the panel with the concentrations of the four different gases monitored by the device. The upper part shows the geolocation of the device. The graphs of the individual concentrations of each gas have been divided into sections. Each section shows the average concentration of the previous half hour, the evolution of the concentration by hour, and the average daily concentration. The device also includes a section displaying the temperature and humidity.

**Figure 11:**
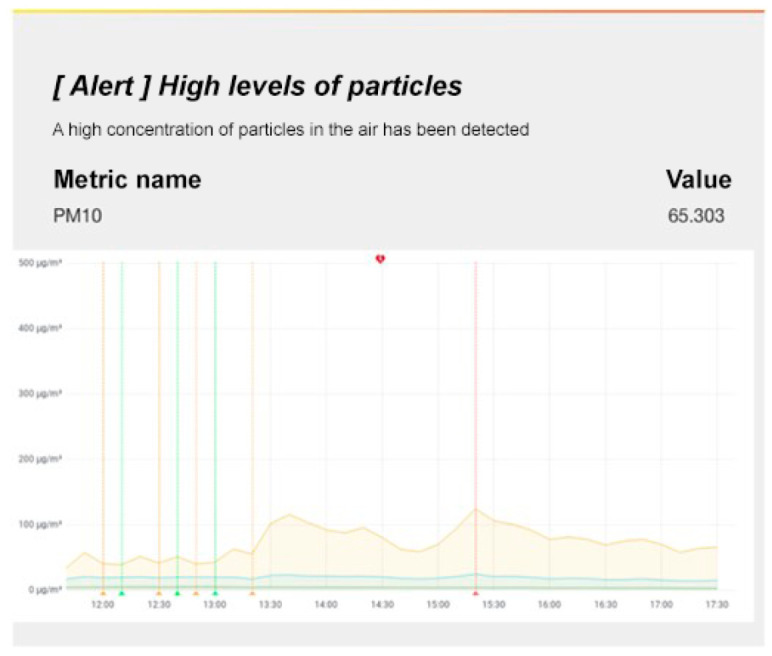
Alert for high PM10 particulate matter levels in the air using the service integrated in Grafana.

Regarding the alerts, Figure 11 shows an example of the alert that is forwarded to a client when the established threshold ( 50 μg/$m^{3}$) is surpassed for an hour. This alert is sent through Grafana, and the evolution of the particulate matter levels over time can be seen in an email.

**Figure 12:**
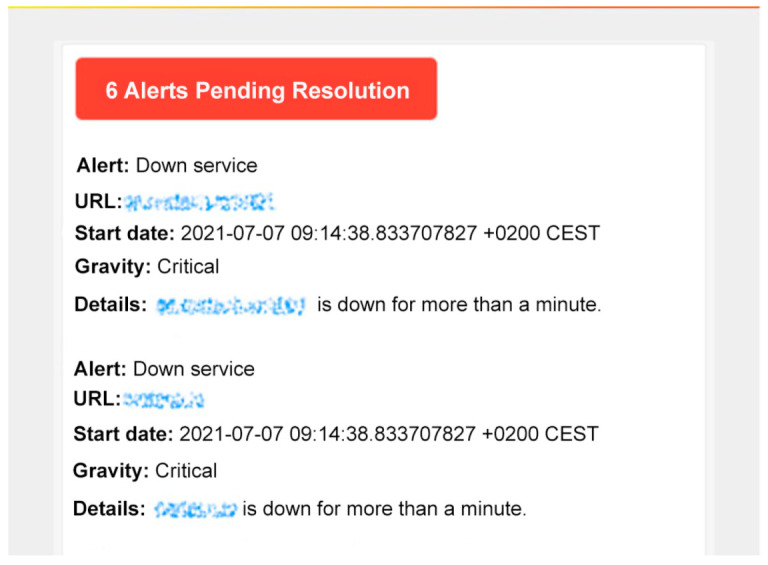
Example of an alert forwarded using *Prometheus AlertManager*.

Figure 12 shows an alert received by the network administrator notifying about a series of crashed services. This alert has been forwarded using the Prometheus *AlertManager* functionality.

The industry may use these alerts to create specific policies and determine the actions to be performed to reduce pollution emissions. Furthermore, as the location of all the deployed devices is known, the source of the pollution is identified by the device that activates the alert.

### 5.2. Machine Learning Techniques Applied to the Environmental Data

The continuous operation of this system for industrial air quality monitoring has generated Big Data that can provide useful insights into the future air quality conditions of the area. In this subsection, various machine learning techniques are applied to the Big Data obtained from the environmental monitoring devices to determine the most accurate algorithm. In the following paragraphs, we detail the methodology used to select the best machine learning technique.

Once the data were collected by the devices, we identified the values considered outliers (unrepresentative values or errors), which were removed from the population (in this work, the sampling values coincide with those of the population). The reason is because, occasionally, sensors can fail in the measurement when capturing particles or the gases under study. To this end, diverse analytical techniques can be applied depending on the sample distribution and the percentage of data to be eliminated. In our case, if the data are treated as separate variables, outliers can be eliminated employing a univariate method. We propose Tukey’s method [41], in which the distribution of a dataset is observed and different regions are identified from their statistical information. To do this, we defined the interquartile range (IQR) as the difference between Q3 (the third quartile or 75th percentile) and Q1 (the first quartile or 25th percentile). Thus, if we want to eliminate extreme values, those greater than Q3 + 3 × IQR and those less than Q1 − 3 × IQR must be removed.

The next step was to separate the data into two groups, denoted as training and test, to derive a good regression model. The cross-validation technique was used, which divides the sample into *k* groups of data. One of them was used for testing and the remaining for training. Once the algorithm was trained and the model obtained, we verified its performance using new data that had not been used in the training process. We used the test data for this. If the error of the test data was much larger than the error of the training data, the model suffered from overfitting that decreased the generality of the test set. In our case, cross-validation was carried out by dividing the sample size into 10 random groups, the last one being the test group.

Finally, several machine learning techniques were selected to process the acquired air quality data and determine which of them made better predictions. Machine learning is a popular solution applied to Big Data obtained from multiple sources to detect and estimate of patterns. In this study, a total of five supervised learning algorithms based on regression were selected, namely Linear Regression, Random Forest Regression, k-nearest neighbors, Support Vector Machine, and Gaussian Process Regression (GPR). The RMSE and R${}^{2}$ metrics were obtained for each technique to determine the most accurate among the tested machine learning techniques.

The statistical details of the datasets gathered by the polluting gas measuring device are presented in Table 3, and the ones for the suspended particulate matter measuring device are presented in Table 4. The polluting gas measuring device gathered over 70,280 observations. The machine learning techniques were checked with 70,000 observations. This number sometimes varied when the outliers were removed. The Gaussian Process Regression technique was tested with 35,000 observations due to the specific processing requirements of this algorithm. The particulate matter measuring device gathered 33,119 observations, which were employed by each of the machine learning techniques under consideration. Tests were performed for the data series acquired from each device without removing the outlier values and once the outlier values were removed. Figures referring to the statistical results presented in Table 3, Table 4 and Table 5 are available in the Appendix A. Finally, note that all the devices were placed in the same location as the official station with the goal of (i) calibrating our devices and (ii) verifying the proper operation of our complete system in real time.

#### 5.2.1. Machine Learning Techniques Applied to the Data from the Polluting Gas Measuring Device

This subsection presents the results of applying the selected machine learning techniques to the data from the polluting gas measuring device.

Table 6 specifies the RMSE and R${}^{2}$ results referring to CO gas for each algorithm. As can be seen, the tests were performed only once due to the absence of outlier values. The Gaussian Process Regression algorithm provided the best results, followed by the Random Forest Regression and the k-nearest neighbors algorithms. For the Gaussian Process Regression algorithm, we used an exponential kernel that adjusted to the dataset better than Random Forest Regression (where the variance reduction is applied as selection criterion from the mean squared error metric), k-nearest Neighbors (based on Euclidean distance), Support Vector Machine (using radial basis function kernel), or Linear Regression algorithms. However, it is important to address the differences in processing times required by these algorithms since the Gaussian Process Regression algorithm required more processing and memory resources than the remaining techniques under consideration. Specifically, the Gaussian Process Regression needed over five hundred times more processing time than Random Forest Regression or k-nearest Neighbors. The Support Vector Machine algorithm was the one offering the worst results, except for the humidity data, being the Linear Regression algorithm the one providing the worst results for all types of data gathered.

**Figure 13:**
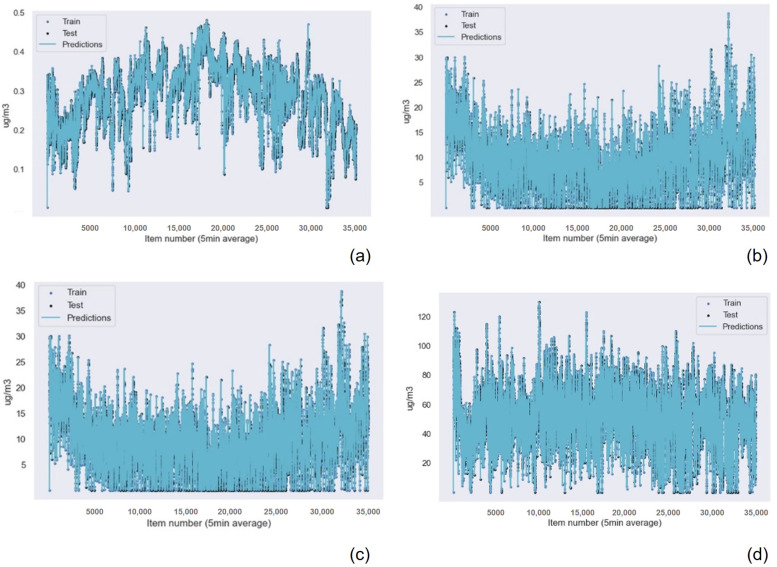
Gaussian Process Regression graphical results for the polluting gas measuring device of (**a**) the CO sensor data, (**b**) the NO${}_{2}$ data with outliers, (**c**) the NO${}_{2}$ data without outliers, and (**d**) the O${}_{3}$ data with outliers.

**Figure 14:**
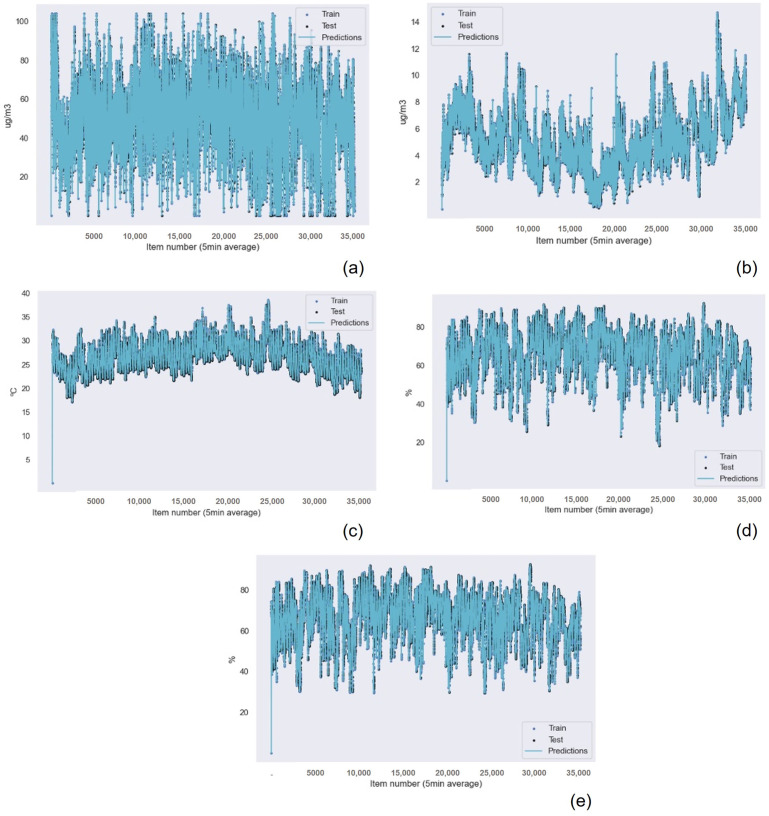
Gaussian Process Regression graphical results for the polluting gas measuring device of (**a**) the O${}_{3}$ data without outliers, (**b**) the SO${}_{2}$ data, (**c**) the temperature data, (**d**) the humidity data with outliers, and (**e**) the humidity data without outliers.

The results for the Gaussian Process Regression algorithm as the best machine learning technique for air quality data were repeated for all the other sensors in the device, as shown in Table 7 for the NO${}_{2}$ data, Table 8 for the O${}_{3}$ data, Table 9 for the SO${}_{2}$ data, Table 10 for the temperature data, and Table 11 for the humidity data. Also, the graphical results for the Gaussian Process Regression technique are represented in Figure 13 and Figure 14. The graphical results for the other machine learning techniques are available in the Appendix A due to space constraints. The Random Forest Regression technique obtains better results than k-nearest neighbors for all the data from all the sensors. However, this difference is minimum if we consider the R${}^{2}$ results. Only the results achieved for the NO${}_{2}$ and O${}_{3}$ sensors presented a greater, but still small, difference in accuracy.

#### 5.2.2. Machine Learning Techniques Applied to the Data from the Suspended Particulate Matter Measuring Device

The results for the suspended particulate matter measuring device are presented in this subsection.

**Figure 15:**
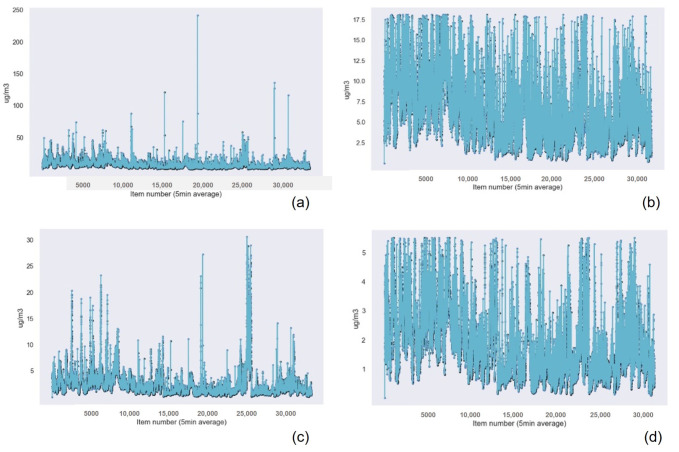
Gaussian Process Regression graphical results for the suspended particulate matter measuring device of (**a**) the PM${}_{2.5}$ sensor data with outliers, (**b**) the PM${}_{2.5}$ data without outliers, (**c**) the PM${}_{1}$ data with outliers, and (**d**) the PM${}_{1}$ data without outliers.

**Figure 16:**
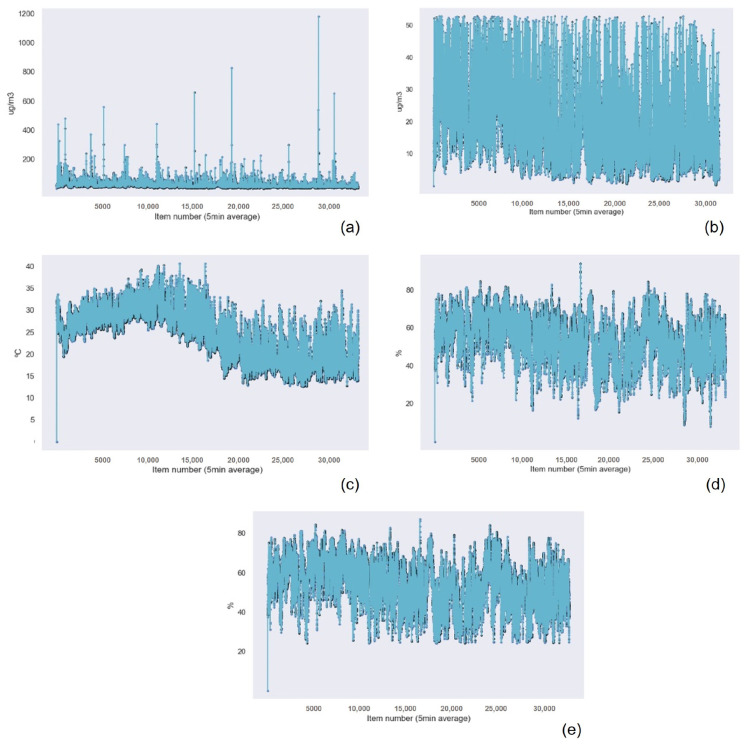
Gaussian Process Regression graphical results for the suspended particulate matter measuring device of (**a**) the PM${}_{10}$ data with outliers, (**b**) the PM${}_{10}$ data without outliers, (**c**) the temperature data, (**d**) the humidity data with outliers, and (**e**) the humidity data without outliers.

Similar to the results obtained for the previous device, the Gaussian Process Regression technique had the best results in terms of accuracy, followed by Random Forest Regression and k-nearest neighbors. Linear Regression presented the worst results for PM${}_{2.5}$, PM${}_{1}$, and humidity data. For the rest of the cases, the worst algorithm was Support Vector Machine. These results are presented in Table 12 for the PM${}_{2.5}$ data, Table 13 for the PM 1 data, Table 14 for the PM 10 data, Table 15 for the temperature data, and Table 16 for the Humidity data. Figure 15 and Figure 16 show the graphical results of the Gaussian Process Regression algorithm for the data captured by the sensor in this device. The rest of the graphical results are available in the Appendix A.

It is important to note that the differences between the results for the Random Forest Regression and k-nearest neighbors are greater than in the previous case. Thus, Random Forest Regression is the best algorithm if there are strict processing time requirements.

## 6. Conclusions and Future Work

A complete air quality monitoring infrastructure for deployment in industries has been presented. Specifically, the proposed system would benefit industries working with soils, stones, grains, or other materials that can produce particulate matter as well as industries susceptible to generate high levels of NO${}_{2}$, O${}_{3}$, SO${}_{2}$, and CO. However, this system can also be extended to other areas of application (smart cities, precision agriculture, smart grids, etc.). Increasingly popular IoT communications technologies, such as LoRa, have been used. Two robust and precise devices have been designed and developed that are able to measure: (i) particulate matter from PM${}_{0.3}$ to PM${}_{40}$ and, (ii) four different gases, SO${}_{2}$, NO${}_{2}$, O${}_{3}$ and CO, which are the main polluting gases in the air according to the WHO. The programming language Python was used to program the controllers of the server, the OPCUA server, and the OPCUA-DA proxy. *MicroPython* was used to program the microcontrollers of the IoT devices. InfluxDB was the database chosen to store all the received data, and Grafana panels were selected to visualize the time series data. An alert system was developed as well. Being aware of environmental pollution levels and when they exceed established limits in real time is vital to proceed with correction interventions, such as halting production processes for a period of time. These alerts can be forwarded through conventional email and by means of instant messaging applications. The development of the infrastructure includes the design of an anti-vandalism casing for both devices, as well as a solar panel able to generate enough power for both devices and charge an auxiliary battery for continuous operation. This facilitates deployment as it provides more flexibility when choosing the location of the facilities.

An intensive study based on machine learning techniques has been carried out to determine the best algorithm to predict trends in future datasets (acquired by the gas/particulate matter devices). These predictions endow our solution with intelligence and activate early alerts, with special emphasis on those that exceed WHO recommended levels.

For future work, we plan to upload the firmware of the devices through OTAA, employing the LoRa communications infrastructure. Creating a mobile app that integrates the Grafana panels, receives alerts, and shows the state of the network with an interface that allows limited modifications to the firmware of the devices, smart dynamic calibration, and predictive maintenance are contemplated as well.

## Figures and Tables

**Table 1:** Maximum particle levels recommended by the WHO.

WHO Recommended Values	Annual Average	24 h Average
PM${}_{2.5}$	10 μg/$m^{-3}$	25 μg/$m^{-3}$
PM${}_{10}$	20 μg/$m^{-3}$	50 μg/$m^{-3}$

**Table 2:** Maximum concentrations of SO${}_{2}$, NO${}_{2}$, and O${}_{3}$ recommended by the WHO.

WHO Recommended Values	Annual Average	24 h Average	8 h Average	Hourly Average	10 min Average
SO${}_{2}$	–	20 μg/$m^{-3}$	–	–	500 μg/$m^{-3}$
NO${}_{2}$	40 μg/$m^{-3}$	–	–	200 μg/$m^{-3}$	–
O${}_{3}$	–	–	100 μg/$m^{-3}$	–	–

**Table 3:** Statistical details of the data for the polluting gas measuring device.

Statistics	CO	NO${}_{2}$	O${}_{3}$	SO${}_{2}$	Temperature	Humidity
Number of observations	70,288	70,288	70,288	70,285	70,300	70,300
Observations used as dataset	30,000 (30,000 GPR)	70,000 (35,000 GPR)	70,000 (35,000 GPR)	70,000 (35,000 GPR)	70,000 (35,000 GPR)	70,000 (69,303 without outliers, 35,000 GPR)
Min value	0.0	0.0	0.0	0.0751	9.35	12.2
Max value	0.481	61.4	130.0	22.4	38.7	94.9
Average	0.178254	17.349788	37.530987	8.697915	21.273490	64.605873
Median	0.177	16.7	37.0	8.34	21.8	65.8
Range	0.481	61.4	130.0	22.3249	29.35	82.7
Variance	0.017707	96.316110	533.894591	18.737172	38.397426	189.958547
Standard deviation	0.133069	9.814077	23.106159	4.328646	6.196566	13.782545
Q1	0.0487	8.94	20.7	4.94	15.5	56.5
Q2	0.177	16.7	37.0	8.34	21.8	65.8
Q3	0.297	25.2	54.1	12.2	26.4	74.5
Outliers (Tukey)	0	85	169	0	0	997

**Table 4:** Statistical details of the data for the particulate matter measuring device.

Statistics	PM${}_{2.5}$	PM${}_{1}$	PM${}_{10}$	Temperature	Humidity
Number of observations	33,119	33,119	33,119	33,119	33,119
Observations used as dataset	33,119 (31,508 without outliers)	33,119 (31,203 without outliers)	33,119 (31,356 without outliers)	33,119 (69,303 without outliers, 33,119 GPR)	33,119 (32,708 without outliers)
Min value	0.17	0.0877	0.208	12.5	7.94
Max value	242.0	30.6	1180.0	40.7	93.9
Average	7.149944	2.259642	21.286887	23.838428	55.414704
Median	5.49	1.59	15.9	24.5	56.6
Range	241.83	30.5123	1179.792	28.2000	85.9600
Variance	36.935864	5.937856	504.650397	34.680362	154.826213
Standard deviation	6.077488	2.436772	22.464425	5.889004	12.442918
Q1	3.2	0.919	9.21	18.1	48.2
Q2	5.49	1.59	15.9	24.5	56.6
Q3	9.19	2.76	26.7	28.6	64.3
Outliers (Tukey)	1611	1916	17.49	0	411

**Table 5:** Statistical details of the particulate matter measuring device # 2.

Statistics	PM${}_{2.5}$	PM${}_{1}$	PM${}_{10}$	Temperature	Humidity
Number of observations	9015	9015	9015	9015	9015
Observations used as dataset	9015 (8303 without outliers)	9015 (8105 without outliers)	9015 (8454 without outliers)	9015 (9007 without outliers)	9015 (8983 without outliers)
Min value	0.0	0.0	0.0	0.0	0.0
Max value	142.0	76.0	332.0	37.9	94.0
Average	7.178333	2.949178	16.880263	21.480355	53.868830
Median	4.81	1.51	12.6	20.7	54.4
Range	142.0	76.0	332.0	37.9	94.0
Variance	62.614450	21.759026	228.405765	27.381352	154.361420
Standard deviation	7.912929	4.664657	15.113099	5.232719	12.424227
Q1	3.0475	0.97375	7.92	17.0	46.0
Q2	4.81	1.51	12.6	20.7	54.4
Q3	8.24	2.91	20.9	25.2	62.8
Outliers (Tukey)	712	906	561	8	32

**Table 6:** CO results.

ML Technique	RMSE	R${}^{2}$
Linear Regression	0.0606371383	0.3457382025
Random Forest	0.0027354389	0.9986685398
k-nearest Neighbors	0.0058150782	0.9939829269
Support Vector Machine	0.0658647137	0.2280666840
Gaussian Process Regression	3.360786$\times 10^{-10}$	1.0

**Table 7:** NO${}_{2}$ results.

	With Outliers		Without Outliers	
ML Technique	RMSE	R${}^{2}$	RMSE	R${}^{2}$
Linear Regression	5.4769488137	0.6876423557	5.3936471548	0.6927516006
Random Forest	0.5663131998	0.9966604485	0.5646376756	0.9966328340
k-nearest Neighbors	1.2744028099	0.9830882630	1.2713923804	0.9829280247
Support Vector Machine	8.9792770734	0.1604288238	8.9049484749	0.1624950419
Gaussian Process Regression	9.334332$\times 10^{-12}$	1.0	9.334332$\times 10^{-12}$	1.0

**Table 8:** O${}_{3}$ results.

	With Outliers		Without Outliers	
ML Technique	RMSE	R${}^{2}$	RMSE	R${}^{2}$
Linear Regression	17.7787994723	0.4072129179	17.5737743925	0.4076389808
Random Forest	1.8993777128	0.9932342407	1.8971387578	0.9930967346
k-nearest Neighbors	4.0697119444	0.9689385869	4.0633023644	0.9683324358
Support Vector Machine	22.2863901849	0.0685204441	22.0219104740	0.0698213572
Gaussian Process Regression	1.794862$\times 10^{-12}$	1.0	1.812172$\times 10^{-12}$	1.0

**Table 9:** SO2 results.

ML Technique	RMSE	R${}^{2}$
Linear Regression	2.4381959621	0.6823210137
Random Forest	0.1537149787	0.9987373481
k-nearest Neighbors	0.3163279069	0.9946528017
Support Vector Machine	3.5221020014	0.3370891440
Gaussian Process Regression	1.780470$\times 10^{-11}$	1.0

**Table 10:** Temperature results.

ML Technique	RMSE	R${}^{2}$
Linear Regression	3.0491692194	0.7579604526
Random Forest	0.0698949997	0.9998728210
k-nearest Neighbors	0.1536363707	0.9993855149
Support Vector Machine	5.3579532919	0.2526550146
Gaussian Process Regression	3.742109$\times 10^{-12}$	1.0

**Table 11:** Humidity results.

	With Outliers		Without Outliers	
ML Technique	RMSE	R${}^{2}$	RMSE	R${}^{2}$
Linear Regression	13.6195399392	0.0242659737	12.7454834625	0.0212357020
Random Forest	0.3295573320	0.99942869433	0.32521124	0.9993627694
k-nearest Neighbors	0.7228922741	0.9972511329	0.7246940306	0.9968357203
Support Vector Machine	13.0700416068	0.1014122056	12.1187865560	0.1151212523
Gaussian Process Regression	1.483299$\times 10^{-12}$	1.0	1.461812$\times 10^{-12}$	1.0

**Table 12:** PM${}_{2.5}$ results for the suspended particulate matter measuring device.

	With Outliers		Without Outliers	
ML Technique	RMSE	R${}^{2}$	RMSE	R${}^{2}$
Linear Regression	5.6339723184	0.1406358736	3.6301232426	0.1995078571
Random Forest	1.0374533951	0.9708603054	0.5031288140	0.9846229611
k-nearest Neighbors	2.2764688104	0.8596956256	1.0622312628	0.9314586755
Support Vector Machine	5.7086327664	0.1177086638	3.4673092396	0.2697030477
Gaussian Process Regression	1.065542$\times 10^{-11}$	1.0	1.342590$\times 10^{-11}$	1.0

**Table 13:** PM${}_{1}$ results for the suspended particulate matter measuring device.

	With Outliers		Without Outliers	
ML Technique	RMSE	R${}^{2}$	RMSE	R${}^{2}$
Linear Regression	2.3789249781	0.0469026363	1.0393676099	0.2139804370
Random Forest	0.1702871285	0.99511640748	0.0980717224	0.9930018604
k-nearest Neighbors	0.3686441913	0.9771129106	0.2101723417	0.9678599814
Support Vector Machine	2.08949638433	0.2647093837	0.59679607853	0.7408525621
Gaussian Process Regression	3.009160$\times 10^{-11}$	1.0	4.645474$\times 10^{-11}$	1.0

**Table 14:** PM${}_{10}$ results for the suspended particulate matter measuring device.

	With Outliers		Without Outliers	
ML Technique	RMSE	R${}^{2}$	RMSE	R${}^{2}$
Linear Regression	21.6415626560	0.07191445240	10.8970611274	0.12145507861
Random Forest	5.6658624623	0.93638744595	2.04753945533	0.96898228817
k-nearest Neighbors	12.177188817	0.70616409871	4.4184046941	0.85556375755
Support Vector Machine	22.5837680622	0.010656531	11.2120912053	0.06992399072
Gaussian Process Regression	3.232586$\times 10^{-12}$	1.0	4.685818$\times 10^{-12}$	1.0

**Table 15:** Temperature results for the suspended particulate matter measuring device.

ML Technique	RMSE	R${}^{2}$
Linear Regression	3.2755739004	0.69075451886
Random Forest	0.15767071532	0.99928347618
k-nearest Neighbors	0.35182904520	0.99643227035
Support Vector Machine	5.08413090899	0.25499016306
Gaussian Process Regression	4.0773021$\times 10^{-12}$	1.0

**Table 16:** Humidity results for the suspended particulate matter measuring device.

	With Outliers		Without Outliers	
ML Technique	RMSE	R${}^{2}$	RMSE	R${}^{2}$
Linear Regression	11.9691770543	0.075222614053	11.38285383117	0.0706829763
Random Forest	0.55854782387	0.99798613967	0.55092812436	0.99782303493
k-nearest Neighbors	1.23655479781	0.99012958638	1.2362885504	0.98903771045
Support Vector Machine	11.72825025083	0.11207748857	11.0564348600	0.1232176516
Gaussian Process Regression	1.764964$\times 10^{-12}$	1.0	1.758820$\times 10^{-12}$	1.0

**Figure A1:**
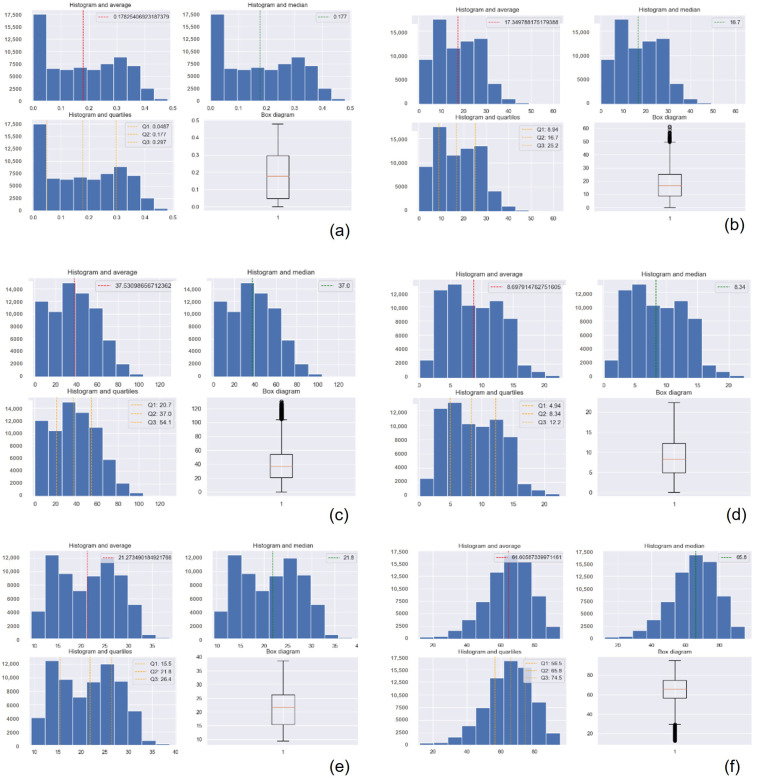
Statistics for (**a**) CO (**b**) NO${}_{2}$ (**c**) O${}_{3}$ (**d**) SO${}_{2}$ (**e**) Temperature and (**f**) Humidity for the polluting gas measuring device.

**Figure A2:**
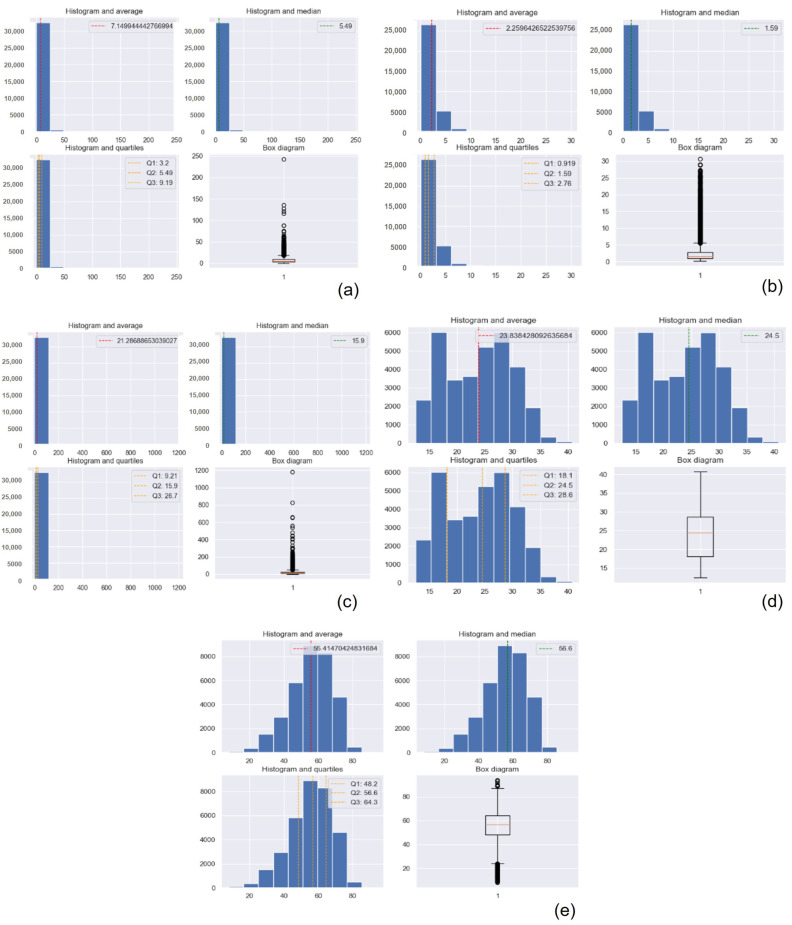
Statistics for (**a**) PM${}_{2.5}$ (**b**) PM${}_{1}$ (**c**) PM${}_{10}$ (**d**) Temperature and (**e**) Humidity for particulate matter measuring device # 1.

**Figure A3:**
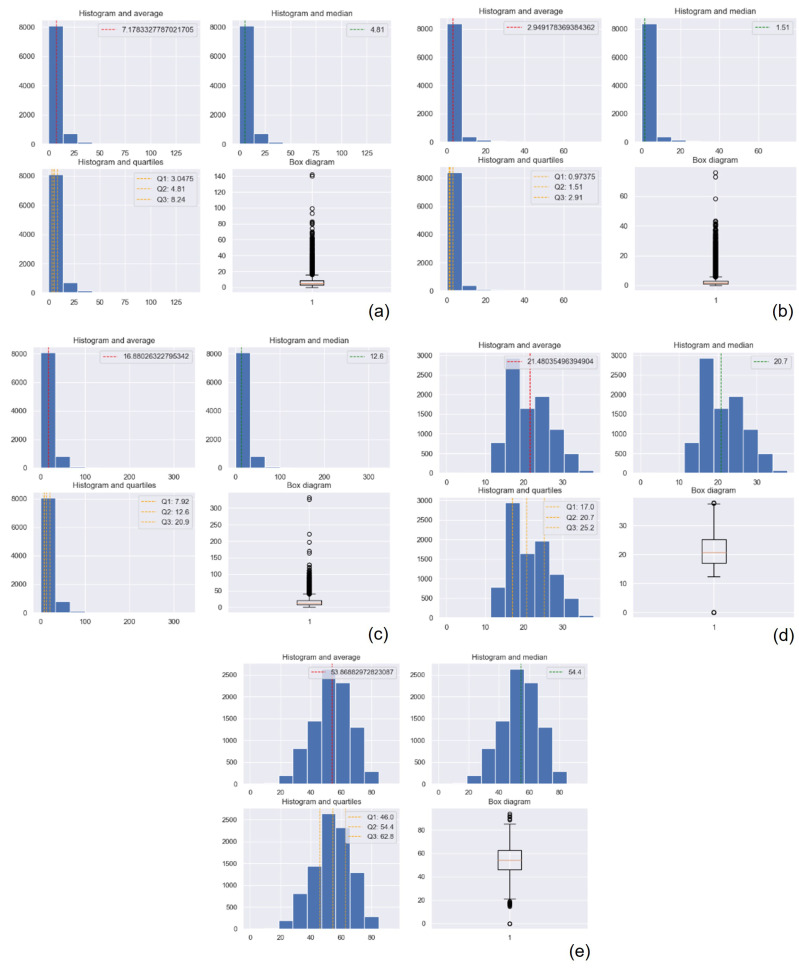
Statistics for (**a**) PM${}_{2.5}$ (**b**) PM${}_{1}$ (**c**) PM${}_{10}$ (**d**) Temperature and (**e**) Humidity for particulate matter measuring device # 2.

**Figure A4:**
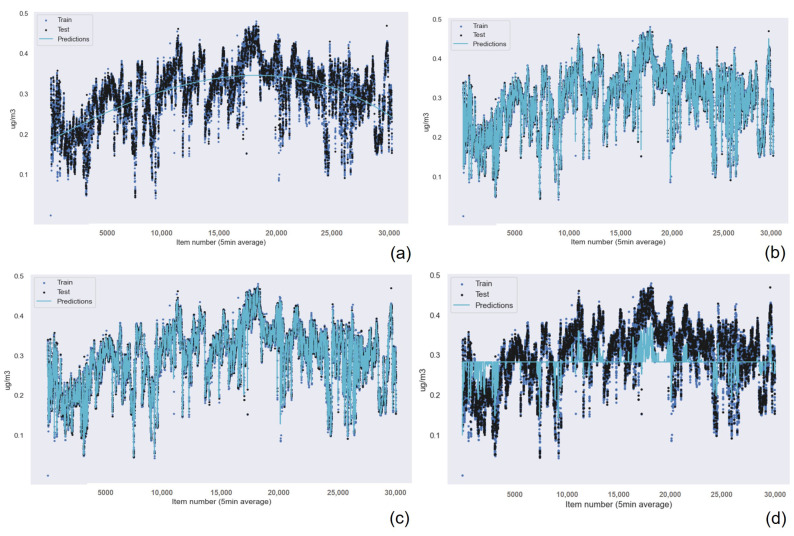
CO results for (**a**) Linear Regression (**b**) Random Forest Regression (**c**) KNN and (**d**) Support Vector Machine.

**Figure A5:**
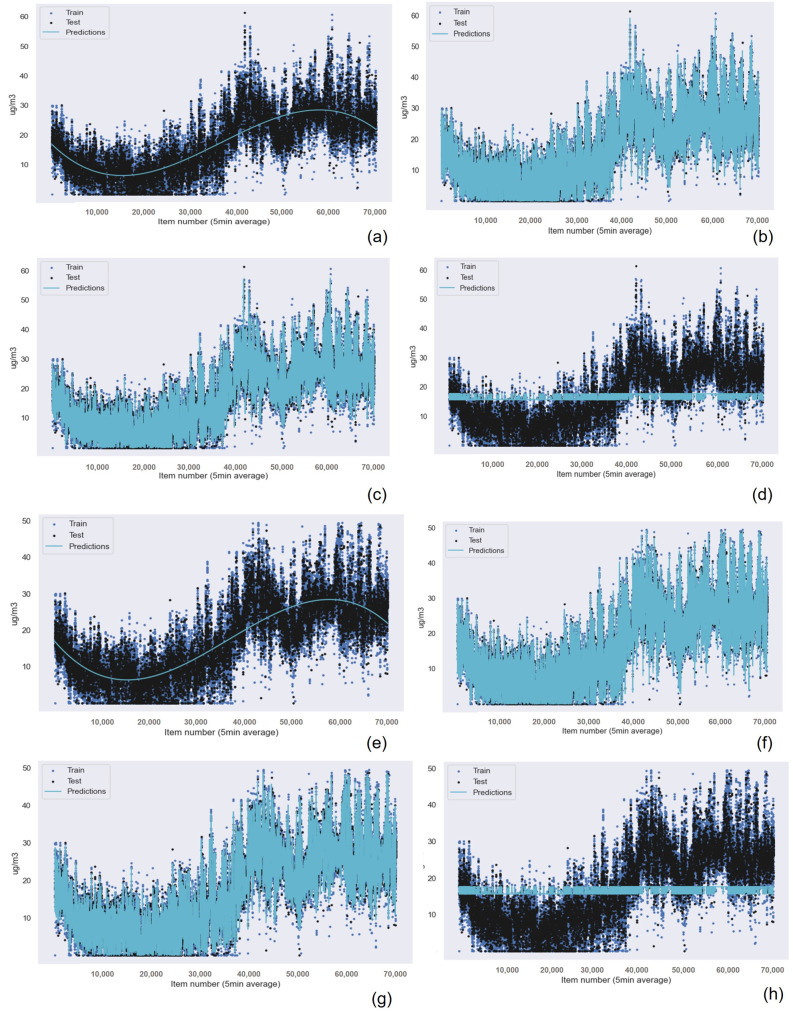
NO${}_{2}$ results with outliers for (**a**) Linear Regression (**b**) Random Forest Regression (**c**) KNN and (**d**) Support Vector Machine, and without outliers for (**e**) Linear Regression (**f**) Random Forest Regression (**g**) KNN and (**h**) Support Vector Machine.

**Figure A6:**
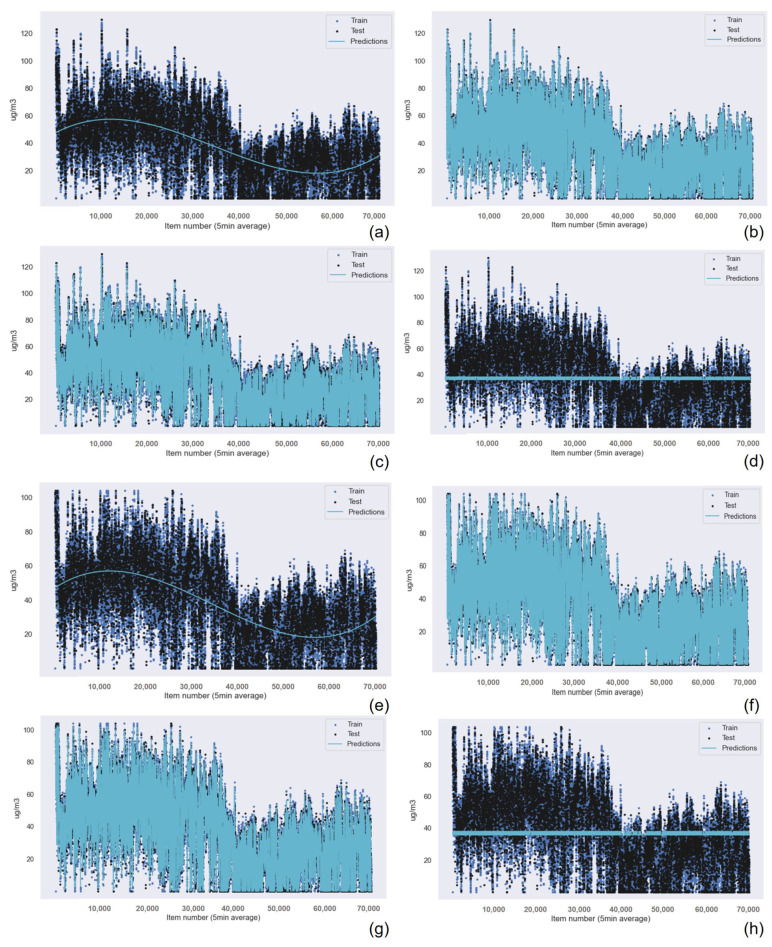
O${}_{3}$ results with outliers for (**a**) Linear Regression (**b**) Random Forest Regression (**c**) KNN and (**d**) Support Vector Machine, and without outliers for (**e**) Linear Regression (**f**) Random Forest Regression (**g**) KNN and (**h**) Support Vector Machine.

**Figure A7:**
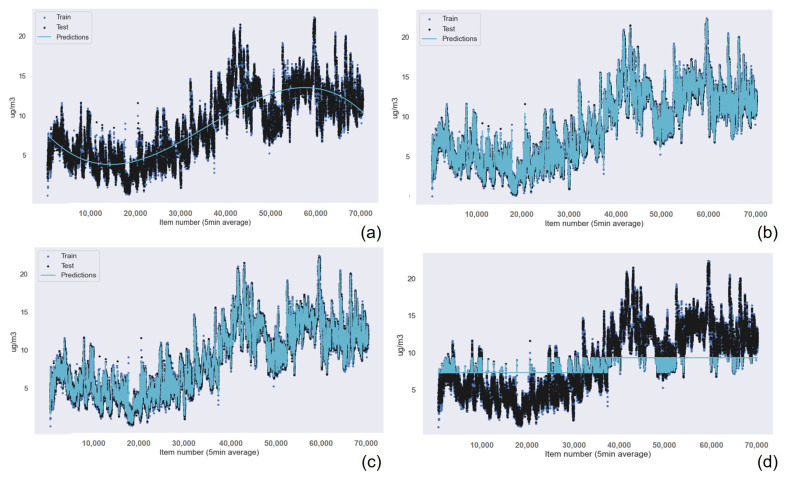
SO${}_{2}$ results for (**a**) Linear Regression (**b**) Random Forest Regression (**c**) KNN and (**d**) Support Vector Machine.

**Figure A8:**
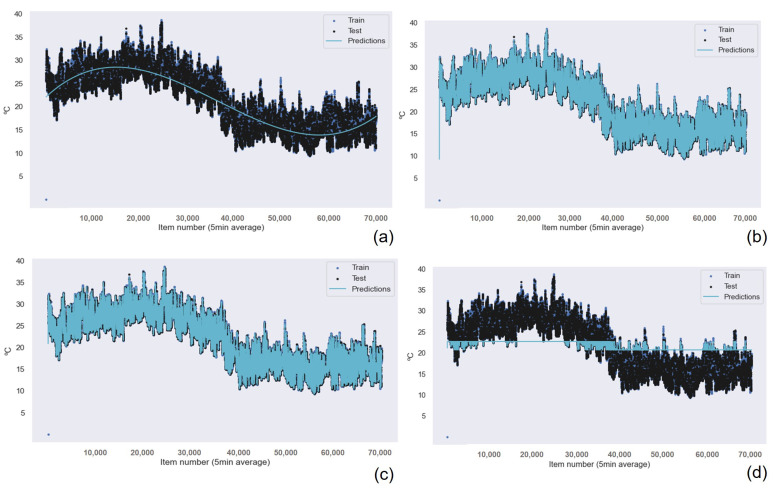
Temperature results for (**a**) Linear Regression (**b**) Random Forest Regression (**c**) KNN and (**d**) Support Vector Machine.

**Figure A9:**
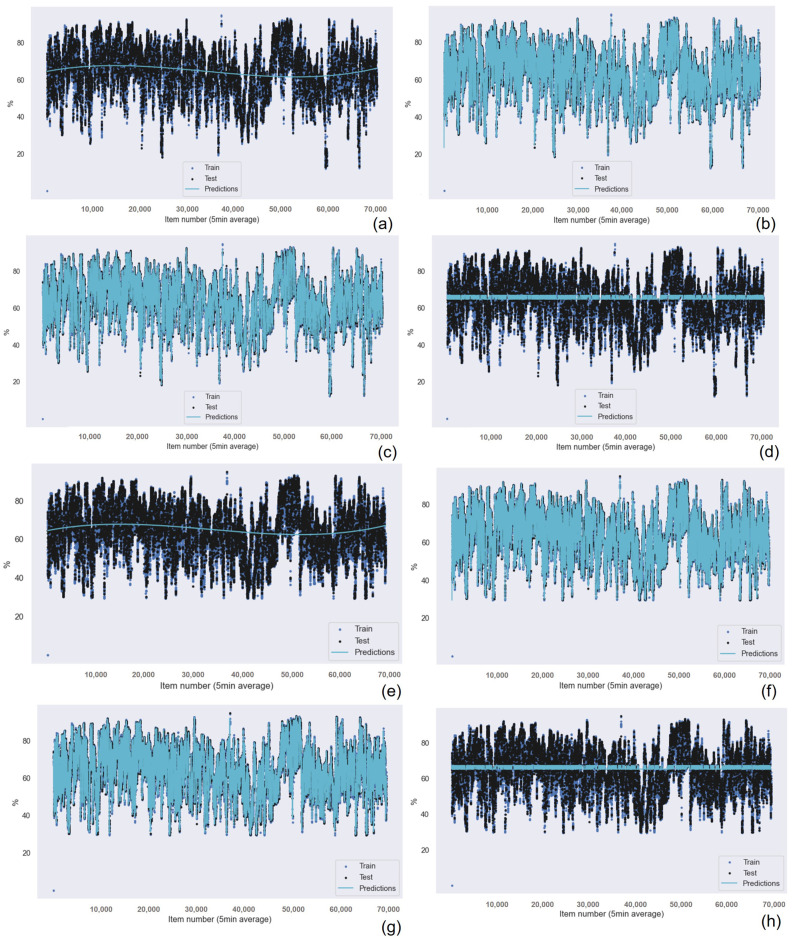
Humidity results with outliers for (**a**) Linear Regression (**b**) Random Forest Regression (**c**) KNN and (**d**) Support Vector Machine, and without outliers for (**e**) Linear Regression (**f**) Random Forest Regression (**g**) KNN and (**h**) Support Vector Machine.

**Figure A10:**
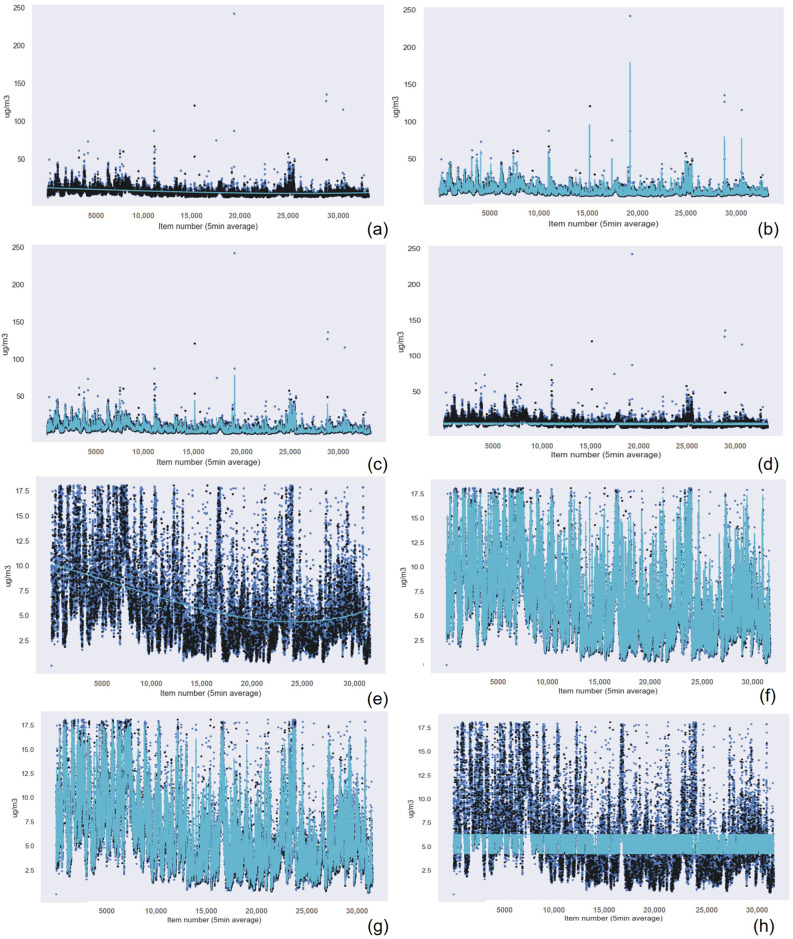
PM${}_{2.5}$ results of the articulate matter measuring device # 1 with outliers for (**a**) Linear Regression (**b**) Random Forest Regression (**c**) KNN and (**d**) Support Vector Machine, and without outliers for (**e**) Linear Regression (**f**) Random Forest Regression (**g**) KNN and (**h**) Support Vector Machine.

**Figure A11:**
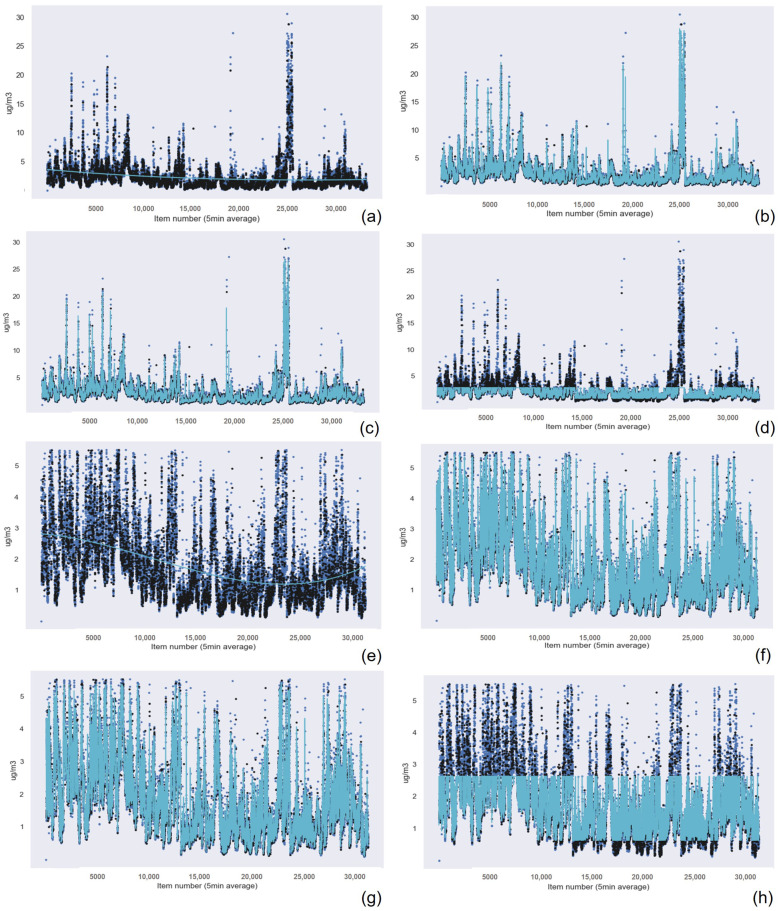
PM${}_{1}$ results of the articulate matter measuring device # 1 with outliers for (**a**) Linear Regression (**b**) Random Forest Regression (**c**) KNN and (**d**) Support Vector Machine, and without outliers for (**e**) Linear Regression (**f**) Random Forest Regression (**g**) KNN and (**h**) Support Vector Machine.

**Figure A12:**
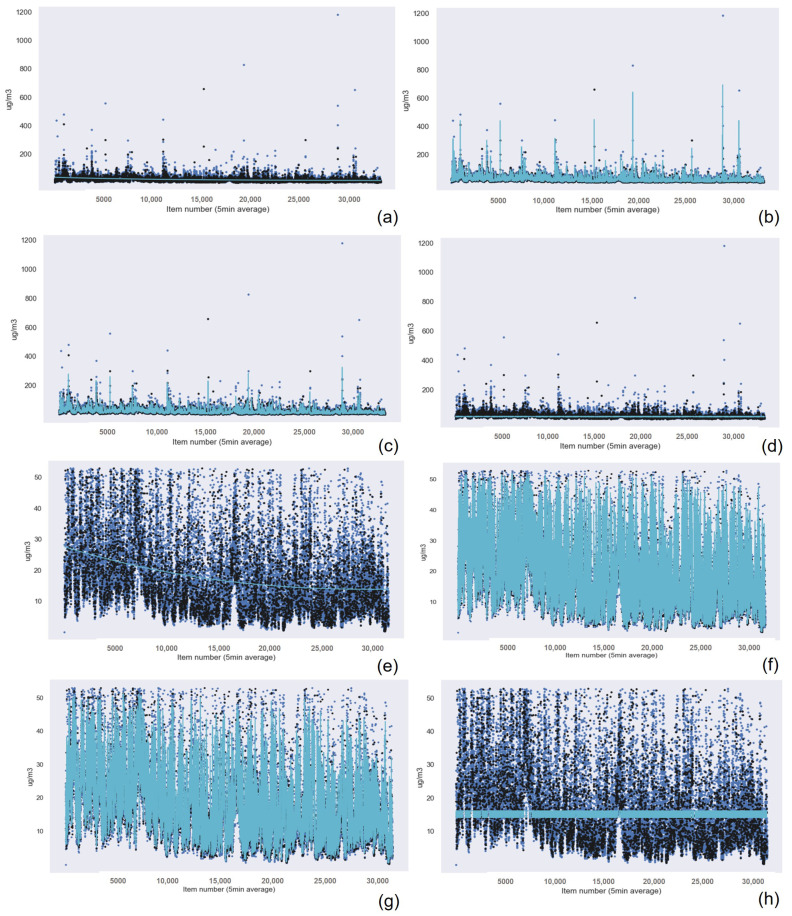
PM${}_{10}$ results of the articulate matter measuring device # 1 with outliers for (**a**) Linear Regression (**b**) Random Forest Regression (**c**) KNN and (**d**) Support Vector Machine, and without outliers for (**e**) Linear Regression (**f**) Random Forest Regression (**g**) KNN and (**h**) Support Vector Machine.

**Figure A13:**
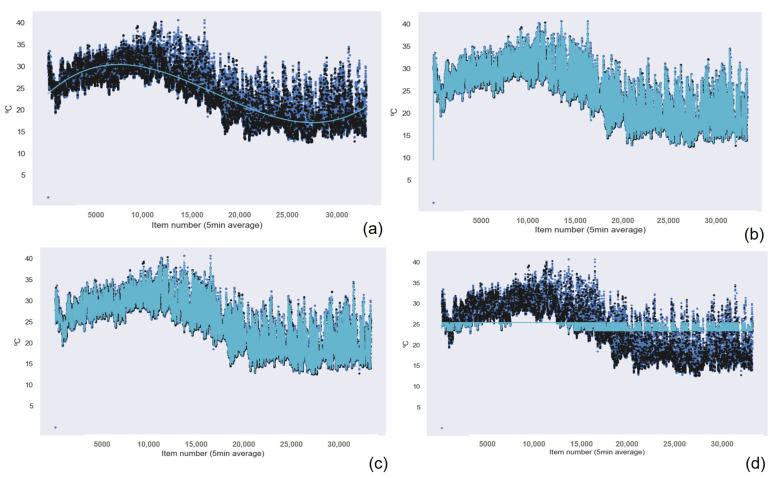
Temperature results for the articulate matter measuring device # 1 for (**a**) Linear Regression (**b**) Random Forest Regression (**c**) KNN and (**d**) Support Vector Machine.

**Figure A14:**
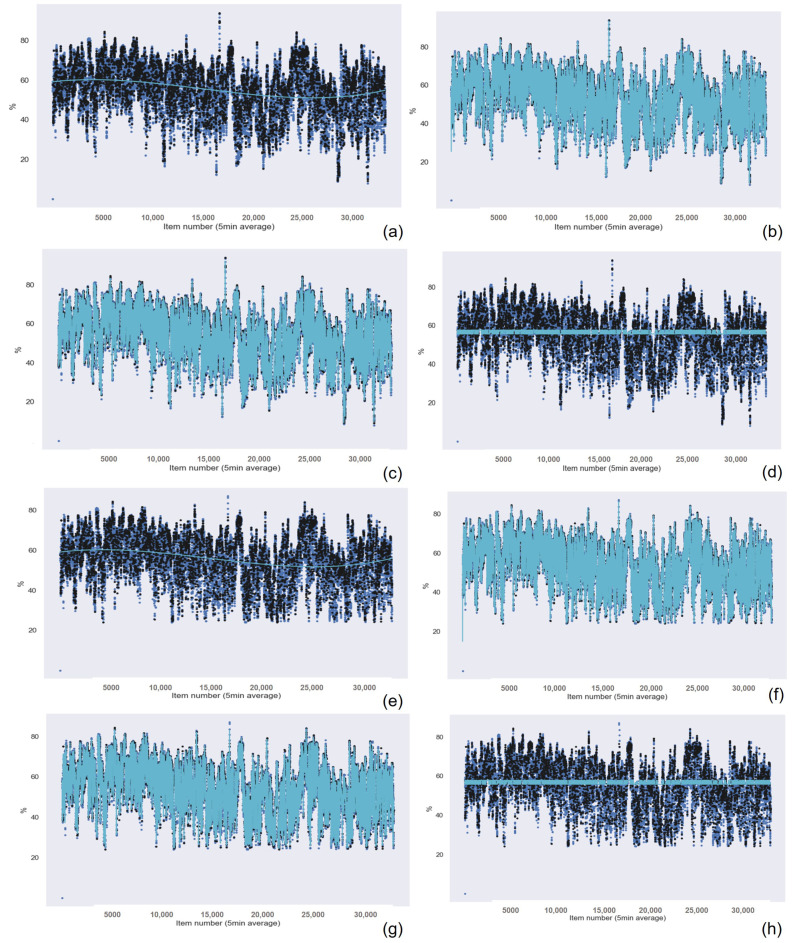
Humidity results of the articulate matter measuring device # 1 with outliers for (**a**) Linear Regression (**b**) Random Forest Regression (**c**) KNN and (**d**) Support Vector Machine, and without outliers for (**e**) Linear Regression (**f**) Random Forest Regression (**g**) KNN and (**h**) Support Vector Machine.

**Figure A15:**
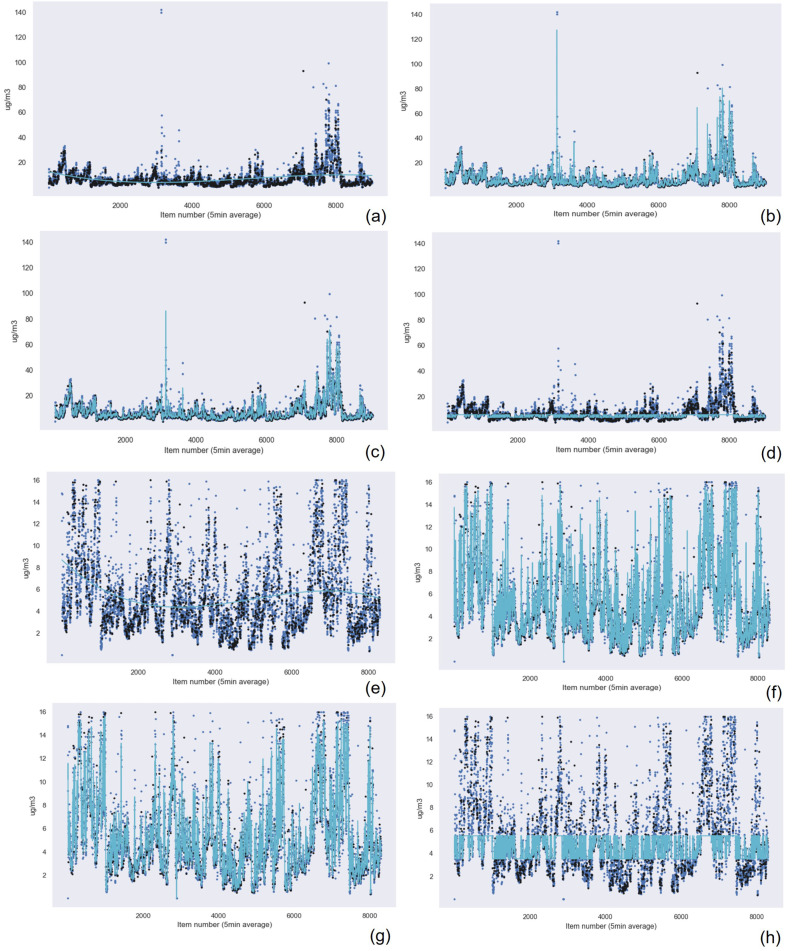
PM${}_{2.5}$ results of the articulate matter measuring device # 2 with outliers for (**a**) Linear Regression (**b**) Random Forest Regression (**c**) KNN and (**d**) Support Vector Machine, and without outliers for (**e**) Linear Regression (**f**) Random Forest Regression (**g**) KNN and (**h**) Support Vector Machine.

**Figure A16:**
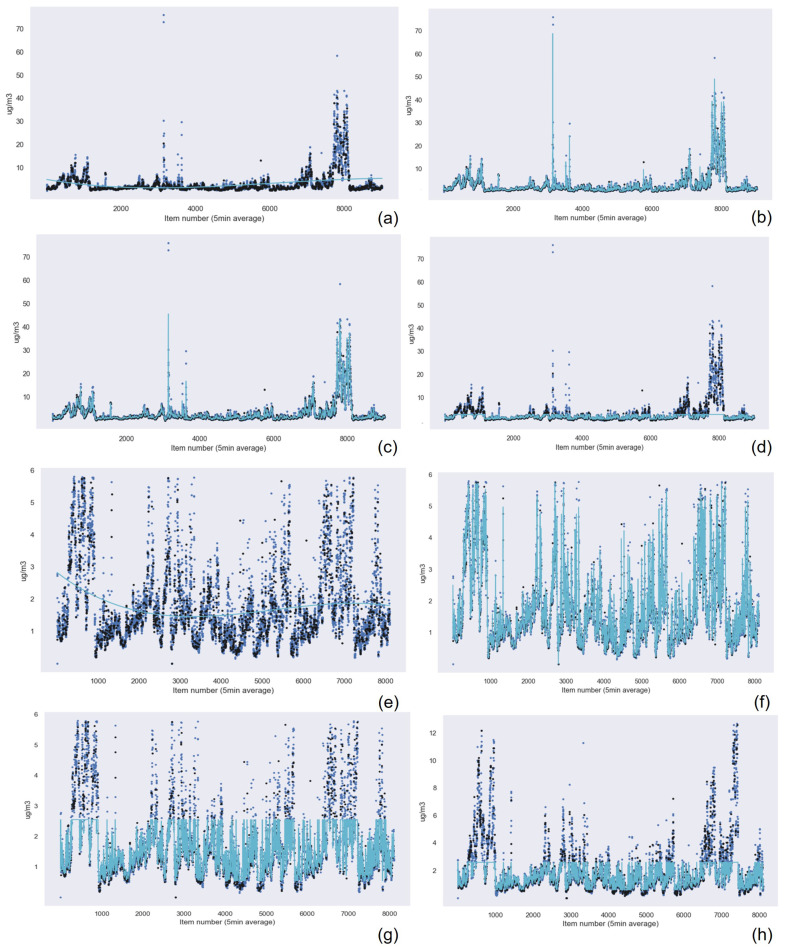
PM${}_{1}$ results of the articulate matter measuring device # 2 with outliers for (**a**) Linear Regression (**b**) Random Forest Regression (**c**) KNN and (**d**) Support Vector Machine, and without outliers for (**e**) Linear Regression (**f**) Random Forest Regression (**g**) KNN and (**h**) Support Vector Machine.

**Figure A17:**
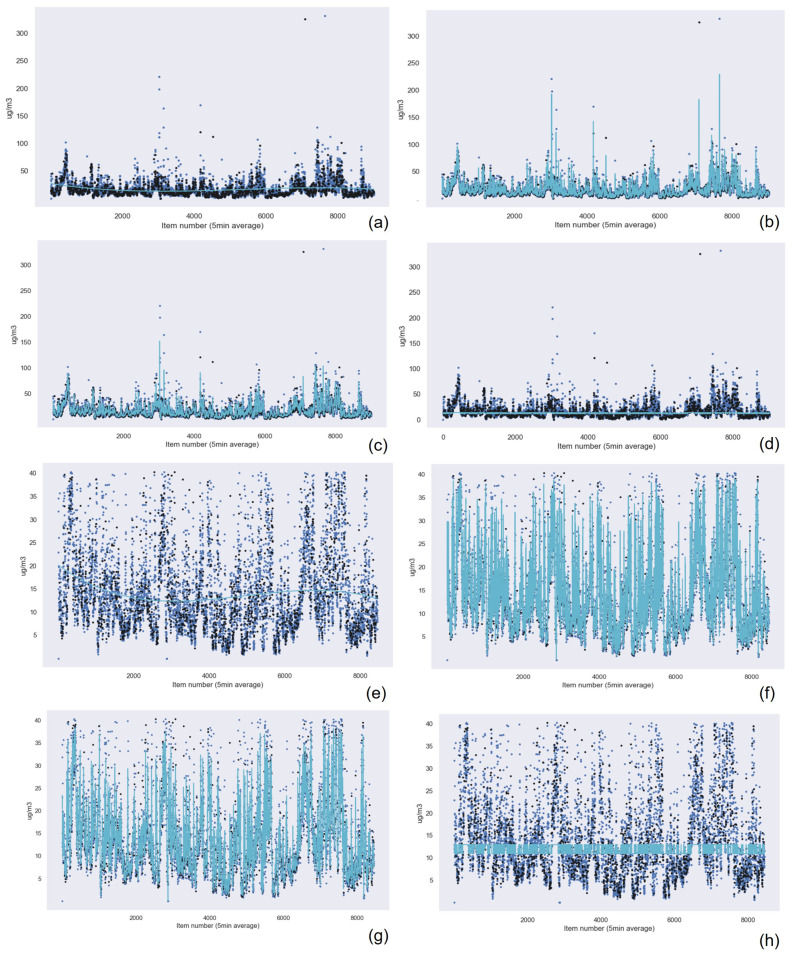
PM${}_{10}$ results of the articulate matter measuring device # 2 with outliers for (**a**) Linear Regression (**b**) Random Forest Regression (**c**) KNN and (**d**) Support Vector Machine, and without outliers for (**e**) Linear Regression (**f**) Random Forest Regression (**g**) KNN and (**h**) Support Vector Machine.

**Figure A18:**
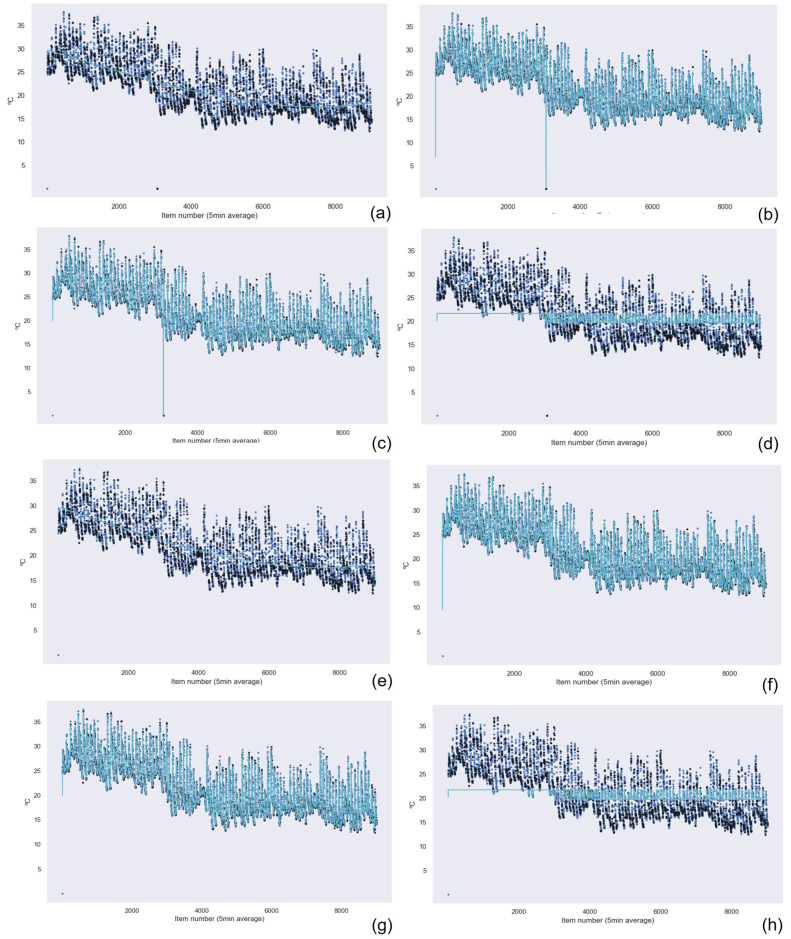
Temperature results of the articulate matter measuring device # 2 with outliers for (**a**) Linear Regression (**b**) Random Forest Regression (**c**) KNN and (**d**) Support Vector Machine, and without outliers for (**e**) Linear Regression (**f**) Random Forest Regression (**g**) KNN and (**h**) Support Vector Machine.

**Figure A19:**
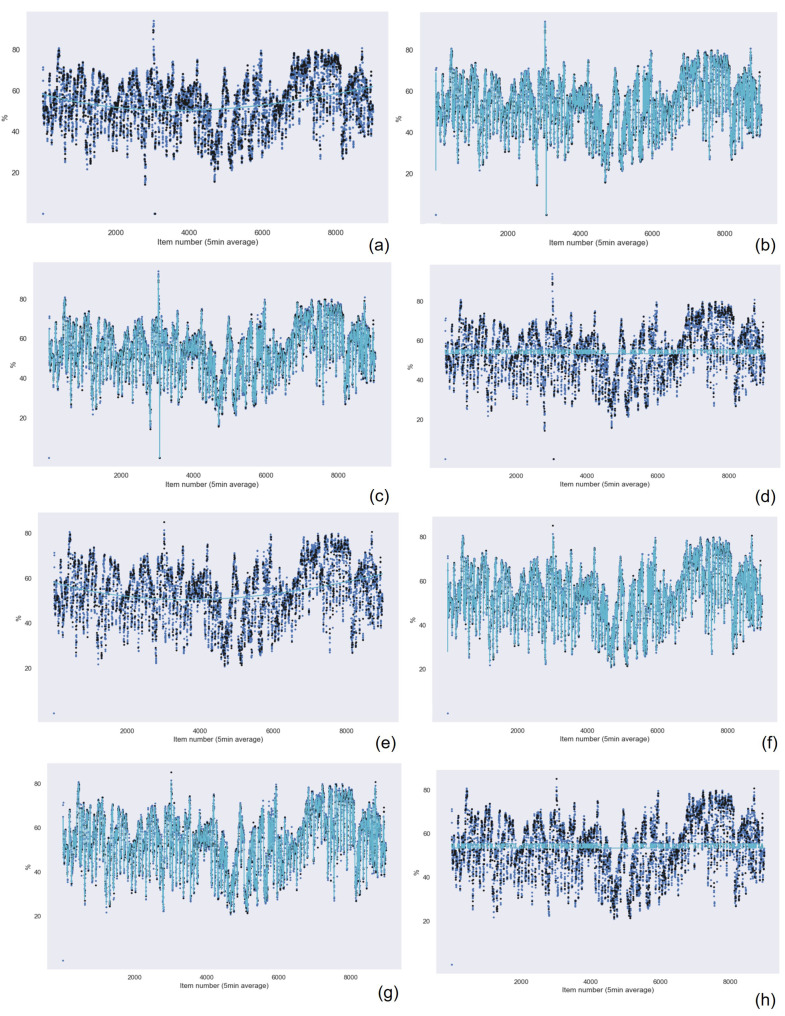
Humidity results of the articulate matter measuring device # 2 with outliers for (**a**) Linear Regression (**b**) Random Forest Regression (**c**) KNN and (**d**) Support Vector Machine, and without outliers for (**e**) Linear Regression (**f**) Random Forest Regression (**g**) KNN and (**h**) Support Vector Machine.

## Data Availability

The data presented in this study are available on request from the corresponding author. The data are not publicly available due to privacy constraints.

## Abbreviations

The following abbreviations are used in this manuscript:

GPS	Global Positioning System
OPC	OLE for Process Control
PLC	Programmable Logic Controller
PLC,[PLCs]	Programmable Logic Controllers
WHO	World Health Organization
VPN	Virtual Private Network
DA	Data Access
UA	Unified Architecture
WHO	World Health Organization
UAV,[UAVs]	Unmanned Aerial Vehicles
LTE	Long Term Evolution

## Appendix A

The data presented in the appendix are the graphical results from the statistical analysis of the data and the Machine-Learning results for each technique applied to the data gathered by all the sensors that comprise the the polluting particle metering devices and the polluting gas measuring device.
